# Longitudinal bidirectional associations among sarcopenia risk, social isolation, and frailty in Chinese older adults using an item response theory-derived frailty index

**DOI:** 10.3389/fpubh.2026.1864444

**Published:** 2026-07-08

**Authors:** Hui-Qian Lin, Ci Lin, Ya-Fen Lin, Xiao-Di Wang, Han-Bing Lin, Xin-Ying Chen, Wu-Xuan Huang, Jin-Lan Zhong, Hong Fan, Ya-Jun Hong

**Affiliations:** 1 Zhongshan Hospital, Fudan University (Xiamen Branch), Xiamen, Fujian, China; 2 State Key Laboratory of Vaccines for Infectious Diseases, Xiang An Biomedicine Laboratory, Xiamen, Fujian, China

**Keywords:** CHARLS, frailty, healthy aging, item response theory, longitudinal analysis, random-intercept cross-lagged panel model, sarcopenia risk, social isolation

## Abstract

**Introduction:**

Frailty is a common geriatric syndrome characterized by multisystem decline and increased vulnerability in older adults, and may interact with sarcopenia risk and social isolation through shared behavioral and functional pathways. However, their longitudinal bidirectional associations remain insufficiently understood. In addition, the frailty index, one of the most widely used tools for frailty assessment, is typically constructed within a conventional equal-weight cumulative-deficit framework and may not fully capture heterogeneity in the contribution of individual deficits.

**Methods:**

This longitudinal observational study used data from the China Health and Retirement Longitudinal Study from 2011 to 2015. A total of 3,159 adults aged 60 years and older with repeated measures of sarcopenia risk, social isolation, and frailty were included. Frailty was assessed using a 40-item item response theory-derived frailty index. Sarcopenia risk was measured using a composite score based on estimated skeletal muscle mass, grip strength, and gait speed. Social isolation was assessed using a multidimensional social isolation index. Longitudinal associations were examined using cross-lagged panel models and random-intercept cross-lagged panel models.

**Results:**

Reciprocal longitudinal associations were observed. At the between-person level, higher sarcopenia risk was associated with higher frailty. At the within-person level, higher sarcopenia risk predicted later social isolation, higher social isolation predicted later frailty, and sarcopenia risk also directly predicted later frailty. Frailty more strongly predicted later social isolation and later sarcopenia risk. Bootstrap-based indirect-effect analyses further suggested that social isolation may represent a modest longitudinal indirect pathway linking sarcopenia risk and frailty, with more consistent evidence for the reverse pathway. No significant subgroup differences were observed by age, sex, education, or residence.

**Conclusion:**

Sarcopenia risk, social isolation, and frailty formed a linked longitudinal process in older adults. Social isolation may represent a modest indirect pathway linking sarcopenia risk and frailty in both temporal directions, with stronger evidence for the reverse pathway. Early identification of both muscle-related vulnerability and social disconnection may help delay frailty progression.

## 1 Introduction

Frailty is a major public health concern in later life. It is commonly defined as a geriatric syndrome characterized by multisystem decline, reduced physiological reserve, and heightened vulnerability to stressors. Frailty is closely associated with a range of adverse health outcomes, including falls, disability, hospitalization, institutionalization, and mortality (1–3). More importantly, frailty is a dynamic process and, to some extent, is preventable and even reversible in its early stages (4). Therefore, early identification of high-risk states and targeted intervention may help delay, attenuate, or even partly reverse frailty progression.

Frailty is also closely related to musculoskeletal health (5, 6). In this context, sarcopenia risk may represent an important upstream factor in frailty progression (5, 7). Sarcopenia is characterized by age-related declines in skeletal muscle mass, muscle strength, and physical performance, and is associated with multiple adverse outcomes in older adults, including falls, fractures, hospitalization, disability, and mortality (8). Sarcopenia and frailty may share several underlying mechanisms, including chronic low-grade inflammation, mitochondrial dysfunction, and hormonal dysregulation (5, 7). Although closely related, sarcopenia and frailty are distinct constructs. Sarcopenia is primarily a muscle-specific condition involving impaired muscle strength, reduced muscle quantity or quality, and poor physical performance. In contrast, frailty reflects vulnerability arising from multisystem decline and the accumulation of health deficits. Accordingly, sarcopenia is commonly regarded as a muscle-specific contributor to the broader frailty process, particularly through physical-function decline (4, 9). Recent longitudinal evidence further suggests that sarcopenia predicts frailty progression and unfavorable frailty transitions (7). Findings from CHARLS also indicate that sarcopenia is independently associated with higher odds of prefrailty and frailty among older Chinese adults (10). However, these studies do not fully explain how within-person increases in sarcopenia risk may translate into subsequent frailty progression over time.

Social isolation may represent a key pathway in this process. Social isolation is generally defined as an objective lack of social ties and social participation (11). Recent longitudinal evidence from CHARLS indicates that social isolation is associated with a higher risk of possible sarcopenia, while persistent social isolation is also associated with an increased risk of incident frailty in older adults (12, 13). In addition, high-quality reviews have shown relatively consistent associations between social vulnerability and frailty, supporting the broader relevance of social disconnection to later-life vulnerability (14). Several mechanisms may help explain these links. Declining muscle function may impair mobility and increase the risk of functional decline in older adults (15). This, in turn, may reduce opportunities for participation in community and social activities. In addition, concerns about falling and activity restriction have been associated with lower social engagement, suggesting that reduced physical capability and confidence may contribute to social withdrawal (16, 17). In turn, social isolation may accelerate frailty progression through reduced physical activity, poorer diet quality, heightened psychological stress, and delayed access to timely support and care (12). At the same time, worsening frailty may further restrict social engagement and increase vulnerability to physical decline, thereby increasing the risk of subsequent sarcopenia. Taken together, social isolation is a plausible pathway linking sarcopenia risk and frailty over time.

Despite increasing recognition of these interconnections, research into the longitudinal bidirectional associations among sarcopenia risk, social isolation, and frailty remains limited. Existing studies generally support the view that these conditions are closely interrelated and may reinforce one another over time. Social vulnerability is closely linked to frailty, social isolation has been associated with later possible sarcopenia, and sarcopenia has been linked to frailty transitions in older adults (14, 18–22). In addition, declining muscle strength may contribute to subsequent reductions in social participation. Recent longitudinal evidence has shown that handgrip strength is positively associated with later formal social engagement, suggesting that poorer muscle function may reduce organizational and community involvement and, potentially, worsen social isolation over time. However, most previous studies have relied on cross-sectional data or pairwise longitudinal analyses, which cannot fully establish longitudinal ordering across all three variables and therefore do not clarify whether social isolation operates as a within-person longitudinal pathway linking sarcopenia risk and frailty (7, 13, 14, 18, 19). To address this gap, longitudinal research is needed to better understand the longitudinal bidirectional associations among sarcopenia risk, social isolation, and frailty.

This issue is particularly important in China. China already has the world’s largest older population, and its ageing process has broad global significance. According to official statistics, older adults in China account for more than one-fifth of the world’s population aged 65 years and above, placing substantial pressure on health and long-term care systems (23). Official projections further indicate that by around 2035, the number of people aged 60 years and above in China will exceed 400 million and account for more than 30% of the total population (24). As population ageing accelerates, the burdens of frailty and related age-associated conditions are likely to increase further. Clarifying the longitudinal bidirectional associations among sarcopenia risk, social isolation, and frailty in Chinese older adults may help identify high-risk groups earlier and support more comprehensive prevention and intervention strategies. This also has important public health implications for promoting healthy ageing and reducing the burden on health and long-term care systems.

Traditional cross-lagged panel models can be used to test bidirectional associations. However, they do not distinguish within-person processes from stable between-person differences and may therefore obscure true intra-individual dynamics (25). To address this issue, we used a random-intercept cross-lagged panel model (RI-CLPM). RI-CLPM separates stable between-person differences from within-person fluctuations over time and provides a more rigorous framework for testing longitudinal directional and pathway effects at the intra-individual level (25, 26). Combining RI-CLPM with an item response theory-derived frailty index may therefore provide a more rigorous and more refined framework for examining longitudinal ordering and the potential pathway linking sarcopenia risk, social isolation, and frailty.

Building on the RI-CLPM framework described above, we used three waves of data from the China Health and Retirement Longitudinal Study to examine the longitudinal associations among sarcopenia risk, social isolation, and frailty in Chinese adults aged 60 years or older. Frailty was measured using an item response theory-derived frailty index. The study was guided by two research questions and corresponding hypotheses. First, we asked whether sarcopenia risk and frailty show longitudinal bidirectional associations at the within-person level. We hypothesized that higher-than-usual sarcopenia risk would predict higher-than-usual frailty at the next wave (Hypothesis 1a), whereas higher-than-usual frailty would predict higher-than-usual sarcopenia risk at the next wave (Hypothesis 1b). Second, we asked whether social isolation acts as a within-person longitudinal pathway linking sarcopenia risk and frailty. We hypothesized that higher-than-usual sarcopenia risk would predict higher-than-usual social isolation at the next wave, which would then predict higher-than-usual frailty at the following wave (Hypothesis 2a). Conversely, higher-than-usual frailty was expected to predict higher-than-usual social isolation at the next wave, which would then predict higher-than-usual sarcopenia risk at the following wave (Hypothesis 2b). Because prior evidence does not clearly indicate whether the forward or reverse pathway should be stronger, we examined these two within-person longitudinal pathways exploratorily.

By integrating an item response theory-derived frailty index with a longitudinal panel design, this study may offer a more nuanced understanding of the longitudinal associations among sarcopenia risk, social isolation, and frailty. The findings may inform integrated strategies for healthy ageing in China and provide evidence relevant to other rapidly ageing populations.

## 2 Methods

### 2.1 Data sources and study population

This study used data from the CHARLS, a nationally representative survey of Chinese residents aged 45 years and older conducted by the National School of Development at Peking University. CHARLS employs a multistage probability sampling design, encompassing over 10,000 households across 150 counties and 450 villages in 28 provinces, and includes assessments of the social, economic, and health circumstances of community residents (27). Information about the survey design and data collection of CHARLS can be obtained from the official website.^1^ The study protocol was approved by the Peking University Institutional Review Board (IRB00001052-11015), and all participants provided written informed consent before the survey. Following the 2011 baseline survey, participants were followed biennially in 2013 (Wave 2), 2015 (Wave 3), 2018 (Wave 4), and 2020 (Wave 5). Data from Wave 1 (2011) to Wave 3 (2015) were used in the present study. Although CHARLS has continued follow-up beyond 2015, the physical examination indicators required for construction of the sarcopenia risk score were available only in the 2011, 2013, and 2015 waves (28). Therefore, analyses were restricted to these three waves to ensure complete longitudinal assessment of all core study variables. Participants were considered eligible for this study if they met the following criteria: (1) were main respondents; (2) were aged 60 years or older; (3) provided repeated measures of the sarcopenia risk score (SRS), social isolation index (SII), and FI from wave 1 to wave 3; and (4) did not have all covariates missing. After exclusion of participants with extreme values, 3,159 participants were included in the final analytic sample. Detailed stepwise information on participant inclusion and exclusion is provided in Supplementary methods S1 and Supplementary Figure S1.

### 2.2 Assessment of sarcopenia risk

In the present study, we used the SRS to assess sarcopenia risk. Drawing on previous research whilst considering data availability, the SRS was constructed from estimated appendicular skeletal muscle mass, grip strength, and gait speed measured at each wave. Estimated appendicular skeletal muscle mass was derived using a validated anthropometric equation. The three components were combined into a continuous score, with higher values indicating greater sarcopenia risk (29). Detailed scoring procedures are provided in the Supplementary methods S3.

### 2.3 Assessment of social isolation

Social isolation was assessed using the SII, adapted from the Berkman–Syme Social Network Index (30). The SII comprised four binary dimensions: marital status, social engagement, organizational participation, and intergenerational contact (31). Scores ranged from 0 to 4, with higher scores indicating greater social isolation. Detailed construction and coding procedures are provided in the Supplementary methods S4.

### 2.4 Assessment of frailty

In the present study, frailty was assessed using an item response theory–derived frailty index (FI-IRT) to improve the precision of frailty measurement at the individual level (32, 33). Frailty was defined as the accumulation of age-related health deficits. Consistent with previous cumulative-deficit frailty index studies and guided by CHARLS data availability (1–3, 34), we selected 40 health-deficit items. These items spanned mobility limitations, activities of daily living, instrumental activities of daily living, chronic diseases, cognitive measures, and self-rated measures, and are detailed in Supplementary Table S3. By design, the SRS and FI-IRT used non-overlapping indicators and different measurement modalities. Estimated appendicular skeletal muscle mass, measured handgrip strength, and measured gait speed were used only to construct the SRS. The FI-IRT was based solely on self-reported health deficits and shared no observed items with the SRS. All items were coded so that higher values indicated greater deficit burden. To balance data completeness and measurement quality, a valid wave-specific frailty assessment required at least 80% non-missing items; assessments with more than 20% missing items were treated as missing and excluded from analysis. The FI-IRT served as the primary frailty measure in the longitudinal analyses. Detailed scoring and psychometric evaluation procedures are provided in Supplementary methods S5.

### 2.5 Covariates

Covariates included sociodemographic characteristics and health-related indicators. Time-invariant sociodemographic covariates assessed at baseline included age, sex, educational attainment, and residence. Time-varying health-related covariates assessed at each wave included depressive symptoms, measured using the 10-item Center for Epidemiologic Studies Depression Scale (CES-D-10) and treated as a continuous variable, as well as drinking status, smoking status, and sleep quality. Detailed definitions and coding of all covariates are provided in the Supplementary methods S6.

### 2.6 Statistical analysis

All analyses were performed in R version 4.4.1. The R packages used for specific statistical procedures are listed in Supplementary methods S8. To ensure data reliability, missing values for the primary analyses were imputed using the missRanger algorithm, a chained random forest imputation method suitable for mixed-type data (35).

After imputation, FI-IRT scores were derived using item response theory (32, 33). Specifically, a unidimensional graded response model (GRM) was fitted separately for each wave using the 40 health-deficit items. The model linked ordered categorical item responses to an underlying latent frailty trait. This approach differs from the conventional deficit-accumulation frailty index, which assigns equal weight to all deficits. The GRM estimated item-specific discrimination and threshold parameters, allowing deficits to contribute differentially to the latent frailty trait. We evaluated the core model assumptions, including unidimensionality and local independence, as well as overall and item-level fit. Each participant’s latent frailty score (*θ*) was then estimated using expected a posteriori scoring and rescaled to a 0–1 metric. Higher values indicated greater frailty. Longitudinal measurement invariance of the FI-IRT was also assessed across the 2011, 2013, and 2015 waves before the scores were entered into the longitudinal models. Details of item coding, GRM estimation, score derivation, model-fit diagnostics, and invariance testing are provided in Supplementary Methods S5.

After all wave-specific scores had been derived, SRS, SII, and FI-IRT values at each of the three waves were screened for extreme values. Screening was applied to derived study scores and was not intended to define clinical abnormality or to classify high-risk frailty, social isolation, or sarcopenia risk as invalid. Consistent with Tukey’s outer-fence rule, values more than three interquartile ranges (IQRs) below the first quartile or above the third quartile of the empirical distribution were classified as extreme outliers (36). This conservative criterion was used to limit the undue influence of highly atypical derived-score values on the covariance structure and parameter estimates of the longitudinal structural models, as outlying observations can affect model parameters and tests in covariance-structure and longitudinal analyses (37, 38). Participants with any extreme outlier at any wave were excluded listwise. This procedure removed 297 participants, yielding a final analytic sample of 3,159. Potential selection bias due to attrition or missing data was assessed by comparing baseline characteristics between included and excluded participants using standardized differences. Details are provided in Supplementary methods S2 and Supplementary Tables S1, S2.

#### 2.6.1 Descriptive statistics and correlation analysis

First, descriptive statistical analyses were conducted to describe the baseline characteristics of the study participants. For continuous variables, means and standard deviations or medians and interquartile ranges were reported as appropriate, and for categorical variables, frequencies and percentages were reported. Second, Spearman correlation analyses were performed to examine the associations among SRS, SII, and FI-IRT both within and across time points. These preliminary associations provided a basis for the subsequent longitudinal modelling.

#### 2.6.2 Longitudinal cross-lagged analyses

CLPM and RI-CLPM were used to explore the longitudinal bidirectional associations among these variables (25, 26). To control for potential confounding and assess the robustness of the observed associations, four sequential models were specified. Model 1 used the CLPM to estimate the longitudinal associations among SRS, SII, and FI-IRT, including autoregressive paths, cross-lagged paths, and within-wave correlations. Model 2 extended Model 1 by additionally adjusting for time-varying covariates (TVCs). Model 3 used the RI-CLPM to estimate within-person associations while accounting for stable between-person differences. Model 4 extended Model 3 by further adjusting for TVCs. TVCs comprised wave-specific health-related and behavioral factors. These included depressive symptoms, drinking status, smoking status, and sleep quality. All were assessed at each survey wave. Time-invariant covariates (TICs) were included in the relevant models as baseline covariates. They represented baseline sociodemographic characteristics, including age, sex, educational attainment, and residence. Because the TVCs may vary over time with changes in lifestyle and social context, their inclusion helped account for within-person time-varying confounding. This modeling strategy allowed us to evaluate the consistency of the observed associations across progressively adjusted models.

Within each model family, temporal stationarity was assessed using a sequence of nested models. We first estimated an unconstrained model and then progressively constrained autoregressive paths, cross-lagged paths, and within-wave residual covariances to equality across waves. All models were estimated using robust maximum likelihood estimation (MLR), which accommodates non-normal data. Model fit was assessed using the Satorra–Bentler scaled *χ*^2^, comparative fit index (CFI), Tucker–Lewis index (TLI), root mean square error of approximation (RMSEA), and standardized root mean square residual (SRMR). Acceptable model fit was defined as CFI or TLI > 0.90, together with RMSEA < 0.08 and SRMR < 0.08. In addition, lower Akaike information criterion (AIC) and Bayesian information criterion (BIC) values indicated better model performance. For nested model comparisons, temporal stationarity was considered acceptable when ΔCFI ≤ 0.010 and ΔRMSEA < 0.015. The most parsimonious acceptable RI-CLPM was retained as the primary model (39, 40).

To further examine longitudinal indirect effects in the primary RI-CLPM, bootstrap-based indirect effects were estimated under the stationarity-constrained primary model. Social isolation was specified as the intervening variable linking sarcopenia risk and frailty in both temporal directions. Indirect effects were quantified as the products of the corresponding cross-lagged path coefficients. Because indirect effects are products of coefficients and typically show asymmetric sampling distributions, their statistical significance was evaluated using nonparametric bootstrap procedures rather than normal-theory tests (41–43). For this analysis, the RI-CLPM was re-estimated using maximum likelihood (ML), and 5,000 bootstrap resamples were used to derive 95% bias-corrected and accelerated (BCa) confidence intervals. Indirect effects were considered statistically significant when the 95% confidence interval excluded zero.

#### 2.6.3 Subgroups and sensitivity analyses

To test the robustness of the findings, several sensitivity analyses were conducted. First, the RI-CLPM was re-estimated using the traditional deficit-accumulation frailty index (FI-DA) instead of the FI-IRT. Second, an alternative frailty parameterization based on log-logistic (*LL*) scaling was examined (44). In community-dwelling older adults, frailty index values are bounded between 0 and 1, typically highly right-skewed, and conceptually unipolar, with many individuals clustered at very low deficit levels and relatively few at the high-frailty end, consistent with evidence that disability and mortality risks increase approximately exponentially as deficits accumulate (45–47). *LL* scaling may therefore provide a more theoretically coherent metric by anchoring the latent trait at zero and stretching the upper tail of the scale. Third, the analyses were repeated using multiple imputation (MI) to evaluate the influence of missing-data handling. Because extreme-value screening was conducted after imputation, the final analytic sample in the MI-based sensitivity analysis could differ slightly from that in the primary analysis. Fourth, the RI-CLPM was re-estimated after excluding participants classified as frail at baseline according to the test characteristic curve-derived FI-IRT threshold. In addition, a multi-group approach was used to test invariance in the RI-CLPMs across age, sex, educational attainment, and residence (40). All statistical analyses were two-sided, and statistical significance was set at *p* < 0.05. Full details of the sensitivity analysis procedures are provided in Supplementary methods S7.

## 3 Results

### 3.1 Characteristics of study participants

Table 1 summarizes the baseline characteristics of the participants. The median SRS was 0.03 (IQR, −0.17 to 0.22), the median SII was 2.00 (IQR, 1.00 to 2.00), and the median FI-IRT was 0.41 (IQR, 0.26 to 0.56). The median CES-D-10 score was 8.00 (IQR, 4.00 to 13.00). Most participants were aged 60–74 years (87.1%), 52.0% were male, 82.7% had an educational attainment below high school, and 84.9% lived in rural areas. In terms of health-related characteristics, 43.7% were current smokers, 31.2% reported alcohol drinking, and 67.0% had good sleep quality. Additionally, the attrition analysis suggested generally comparable baseline profiles between included and excluded participants, with detailed results provided in Supplementary Tables S1, S2.

**Table 1:** Participant characteristics reported or measured at baseline (*n* = 3,159).

Variable	*n* (%) or median (IQR)
Total sample	3,159
Main study variables	
SRS	0.03 (−0.17, 0.22)
SSI	2.00 (1.00, 2.00)
FI-IRT	0.41 (0.26, 0.56)
Baseline covariates	
*Time-invariant covariates*	
Age (years)	
60–74	2,751 (87.1)
≥75	408 (12.9)
Gender	
Female	1,516 (48.0)
Male	1,641 (52.0)
Education	
Below high school	2,612 (82.7)
High school and above	545 (17.3)
Residence	
Urban	478 (15.1)
Rural	2,681 (84.9)
*Baseline time-varying covariates*	
Depression symptom (CES-D-10)	8.00 (4.00, 13.00)
Smoking	
No	1778 (56.3)
Yes	1,381 (43.7)
Drinking	
No	2,172 (68.8)
Yes	987 (31.2)
Sleep quality	
Good	2,115 (67.0)
Poor	1,044 (33.0)

Continuous variables are presented as median (Q_1_, Q_3_), and categorical variables as *n* (%). SRS, Sarcopenia Risk Score; SII, Social Isolation Index; FI-IRT, item response theory-derived frailty index. Depression was assessed using the 10-item Center for Epidemiologic Studies Depression Scale.

### 3.2 Correlation among sarcopenia risk, social isolation, and frailty

Spearman correlation coefficients for SRS, SII, and FI-IRT across the three waves (2011, 2013, and 2015) showed that each variable was significantly positively correlated over time, with frailty demonstrating the highest temporal stability (see Supplementary Table S8). Specifically, SRS showed moderate temporal stability across waves (*r* = 0.48–0.59, all *p* < 0.001), SII showed modest but consistent temporal stability (*r* = 0.42–0.46, all *p* < 0.001), and FI-IRT demonstrated the strongest temporal stability (*r* = 0.58–0.64, all *p* < 0.001).

### 3.3 Model fit of the longitudinal analyses

Before fitting the longitudinal panel models, we assessed the cross-wave measurement comparability of the FI-IRT using an IRT-based longitudinal measurement invariance analysis with a multiple-group GRM. The results supported metric invariance and partial scalar invariance, indicating acceptable longitudinal comparability of the FI-IRT across survey waves. Details of this supplementary analysis are provided in Supplementary methods S5.3. Table 2 summarizes the fit indices for the nested CLPM and RI-CLPM specifications. Results indicate that the CLPM with TICs demonstrated relatively poor fit. Specifically, Model 1a showed poor fit (CFI = 0.917, TLI = 0.334, RMSEA = 0.166, SRMR = 0.033). Models 1b and 1c showed slight improvement, but both exceeded the prespecified cutoff for change in fit and were therefore rejected. Model 1d was acceptable according to the nested comparison criteria, but its overall fit remained unsatisfactory (CFI = 0.914, TLI = 0.705, RMSEA = 0.111, SRMR = 0.035).

**Table 2:** Model fit indices for nested stationarity tests across CLPM and RI-CLPM specifications.

Models	*χ*^2^ (*df*)	RMSEA [90% CI]	CFI	TLI	SRMR	AIC	BIC	Δ*df*	ΔCFI	ΔRMSEA	Decision
CLPM with time-invariant covariates (TIC)											
Model 1a	575.16*** (9)	0.166 [0.156, 0.176]	0.917	0.334	0.033	25839.56	26415.07	—	—	—	—
Model 1b	630.37*** (12)	0.145 [0.136, 0.153]	0.916	0.496	0.033	25844.17	26401.51	3	0.001	**0.022** ^**†**^	Reject
Model 1c	679.89*** (18)	0.119 [0.112, 0.126]	0.915	0.660	0.034	25847.18	26368.17	6	0.001	**0.026** ^**†**^	Reject
Model 1d*^a^*	701.69*** (21)	0.111 [0.104, 0.117]	0.914	0.705	0.035	25854.81	26357.63	3	0.001	0.008	Accept
CLPM with TIC and time-varying covariates (TVC)											
Model 2a	921.75*** (93)	0.055 [0.052, 0.058]	0.920	0.845	0.024	109083.71	110640.62	—	—	—	—
Model 2b	931.29*** (96)	0.054 [0.051, 0.057]	0.920	0.849	0.024	109084.74	110623.47	3	<0.001	0.001	Accept
Model 2c	942.05*** (102)	0.053 [0.050, 0.056]	0.919	0.857	0.024	109084.09	110586.47	6	<0.001	0.001	Accept
Model 2d	954.87*** (105)	0.052 [0.049, 0.055]	0.918	0.860	0.024	109092.57	110576.78	3	0.001	<0.001	Accept
RI-CLPM with time-invariant covariates (TIC)											
Model 3a	64.85*** (27)	0.021 [0.014, 0.028]	0.996	0.989	0.010	25074.14	25540.61	—	—	—	—
Model 3b	69.38*** (30)	0.020 [0.014, 0.027]	0.996	0.990	0.011	25073.06	25521.36	3	<0.001	0.001	Accept
Model 3c	78.21*** (36)	0.019 [0.013, 0.025]	0.996	0.991	0.012	25069.90	25481.84	6	<0.001	0.001	Accept
Model 3d*^a^*	99.37*** (39)	0.022 [0.017, 0.028]	0.994	0.988	0.014	25085.94	25479.71	3	0.002	0.003	Accept
RI-CLPM with TIC and time-varying covariates (TVC)											
Model 4a	462.84*** (111)	0.032 [0.029, 0.035]	0.968	0.948	0.027	108533.72	109981.59	—	—	—	—
Model 4b	466.23*** (114)	0.031 [0.028, 0.034]	0.968	0.949	0.027	108531.34	109961.04	3	<0.001	<0.001	Accept
Model 4c	471.61*** (120)	0.031 [0.028, 0.034]	0.968	0.952	0.027	108524.71	109918.05	6	<0.001	0.001	Accept
Model 4d*^b^*	482.70*** (123)	0.031 [0.028, 0.033]	0.967	0.952	0.027	108531.01	109906.18	3	0.001	<0.001	Accept

CLPM, cross-lagged panel model; RI-CLPM, random-intercept cross-lagged panel model; TIC, time-invariant covariates; TVC, time-varying covariates. Models 1–2 = traditional CLPM; Models 3–4 = RI-CLPM. Nested stationarity tests: **(a)** unconstrained; **(b)** autoregressive (AR) paths constrained equal across waves; **(c)** AR and cross-lagged (CL) paths constrained equal; **(d)** full stationarity with AR, CL, and residual covariances constrained equal. ΔCFI and ΔRMSEA were evaluated against the preceding nested model; ΔCFI ≤ 0.010 and ΔRMSEA < 0.015 indicated that imposing constraints did not meaningfully worsen model fit.

*^a^* RI-CLPM with TIC (Model 3d) retained as the best-fitting parsimonious model (lowest BIC).

*^b^* Primary analysis model: RI-CLPM with both TIC and TVC, providing the most comprehensive covariate adjustment; selected for within-person inference.

^†^Exceeded the prespecified cutoff for change in fit (ΔCFI > 0.010 and/or ΔRMSEA ≥ 0.015). ***p < 0.001.

By contrast, the CLPM with TICs and TVCs showed substantially improved fit. Model 2a was acceptable (CFI = 0.920, TLI = 0.845, RMSEA = 0.055, SRMR = 0.024), and Models 2b–2d remained acceptable under progressively stronger constraints, suggesting that adjustment for covariates improved model performance.

The RI-CLPM with TICs exhibited further enhancement in fit. Model 3a demonstrated excellent fit (CFI = 0.996, TLI = 0.989, RMSEA = 0.021, SRMR = 0.010), and Models 3b–3d all remained acceptable. Among them, Model 3d showed the lowest BIC and was therefore retained as the best-fitting parsimonious model for examining the between-person effects of SRS, SII, and FI-IRT.

The RI-CLPM with TICs and TVCs also demonstrated good fit. Model 4a showed good fit (CFI = 0.968, TLI = 0.948, RMSEA = 0.032, SRMR = 0.027), and Models 4b–4d remained acceptable under progressively stronger stationarity constraints. The final constrained model (i.e., Model 4d) exhibited a good model fit and a nonsignificant difference relative to Models 4a–4c. All fit indices for Model 4d met the recommended criteria (CFI = 0.967, TLI = 0.952, RMSEA = 0.031, SRMR = 0.027). Furthermore, Model 4d incorporated the most comprehensive covariate adjustment and showed relatively low AIC and BIC values. Therefore, Model 4d was selected to examine the within-person effects of SRS, SII, and FI-IRT.

### 3.4 Longitudinal associations among sarcopenia risk, social isolation, and frailty

Supplementary Table S9 presents the standardized coefficients for autoregressive paths, cross-lagged paths, and synchronous correlations among SRS, SII, and FI-IRT across the four best-fitting panel models examining their associations in Chinese older adults. In the CLPM with TICs and TVCs (Model 2d), SII significantly predicted SRS from *T*_1_ to *T*_2_ (*β* = 0.065, *p* < 0.001) and from *T*_2_ to *T*_3_ (*β* = 0.069, *p* < 0.001). SRS also demonstrated significant predictive effects on SII from *T*_1_ to *T*_2_ (*β* = 0.071, *p* < 0.001) and from *T*_2_ to *T*_3_ (*β* = 0.069, *p* < 0.001). Similarly, SRS showed significant positive predictive effects on FI-IRT from *T*_1_ to *T*_2_ (*β* = 0.071, *p* < 0.001) and from *T*_2_ to *T*_3_ (*β* = 0.072, *p* < 0.001), and FI-IRT significantly predicted subsequent SRS from *T*_1_ to *T*_2_ (*β* = 0.075, *p* < 0.001) and from *T*_2_ to *T*_3_ (*β* = 0.076, *p* < 0.001). In contrast, the longitudinal association between FI-IRT and SII was relatively weaker. During the period from *T*_1_ to *T*_2_, SII did not significantly predict FI-IRT (*β* = 0.007, *p* = 0.463), and FI-IRT also did not significantly predict SII (*β* = 0.022, *p* = 0.057). During the period from *T*_2_ to *T*_3_, neither SII nor FI-IRT showed a significant predictive effect on the other (*β* = 0.007, *p* = 0.464; *β* = 0.022, *p* = 0.058).

**Figure 1:**
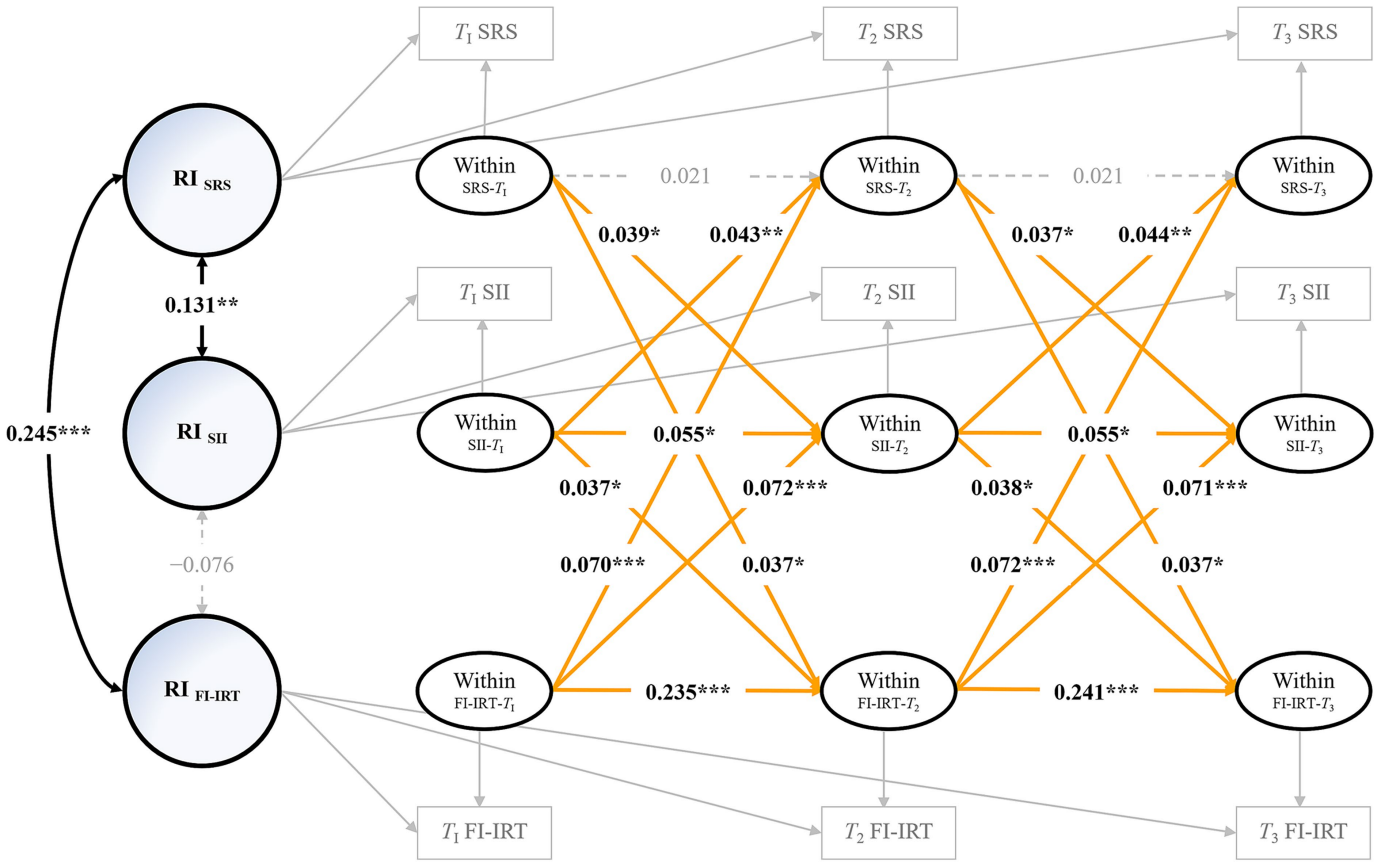
Random-intercept cross-lagged panel model of sarcopenia risk, social isolation, and frailty among Chinese older adults, CHARLS, 2011–2015 (*n* = 3,159). Shown is the primary RI-CLPM with time-invariant and time-varying covariates (Model 4d). For clarity, covariates, residual correlations, and other estimated model components were included in the analysis but are not fully displayed in the diagram. The model was adjusted for age, sex, educational attainment, residence, depressive symptoms, smoking status, drinking status, and sleep quality. RI_SRS_, RI_SII_, and RI_FI-IRT_ denote the random intercepts of the sarcopenia risk score (SRS), social isolation index (SII), and item response theory-derived frailty index (FI-IRT), respectively. *T*_1_, *T*_2_, and *T*_3_ denote Waves 1, 2, and 3, respectively. Standardized coefficients are shown next to the paths. Significant paths are shown as solid orange lines, and non-significant paths are shown as dashed gray lines. Between-person correlations among the random intercepts are displayed on the left. *p < 0.05, **p < 0.01, ***p < 0.001.

After accounting for individual heterogeneity, the RI-CLPM (Model 4d) revealed a distinctly different pattern of associations (see Figure 1). The bidirectional association between SRS and SII remained statistically significant, although the effect sizes became relatively weaker. Specifically, SII significantly predicted SRS from *T*_1_ to *T*_2_ (*β* = 0.043, *p* = 0.003) and from *T*_2_ to *T*_3_ (*β* = 0.044, *p* = 0.003), whereas SRS significantly predicted SII from *T*_1_ to *T*_2_ (*β* = 0.039, *p* = 0.014) and from *T*_2_ to *T*_3_ (*β* = 0.037, *p* = 0.013). Similarly, the association between SRS and FI-IRT showed an attenuated trend. From *T*_1_ to *T*_2_, SRS significantly predicted FI-IRT (*β* = 0.037, *p* = 0.025), and FI-IRT significantly predicted SRS (*β* = 0.070, *p* < 0.001). During the period from *T*_2_ to *T*_3_, SRS positively predicted FI-IRT (*β* = 0.037, *p* = 0.025), and FI-IRT predicted SRS (*β* = 0.072, *p* < 0.001). In contrast to the attenuated associations described above, the bidirectional association between FI-IRT and SII emerged in the RI-CLPM. From *T*_1_ to *T*_2_, SII positively predicted FI-IRT (*β* = 0.037, *p* = 0.030), and FI-IRT predicted SII (*β* = 0.072, *p* < 0.001). This bidirectional association persisted from *T*_2_ to *T*_3_, with SII predicting FI-IRT (*β* = 0.038, *p* = 0.029) and FI-IRT predicting SII (*β* = 0.071, *p* < 0.001). Furthermore, the RI-CLPM identified significant between-person correlations among the random intercepts, with the strongest latent trait correlation observed between SRS and FI-IRT (*β* = 0.245, *p* < 0.001), followed by SRS and SII (*β* = 0.131, *p* = 0.001). In contrast, the correlation between SII and FI-IRT was not statistically significant (*β* = −0.076, *p* = 0.272). Bootstrap-based tests of longitudinal indirect effects in the primary RI-CLPM supported both temporal directions (Supplementary Table S16). In the primary model, a small forward indirect effect from SRS to later FI-IRT via SII was identified (*B* = 0.0008, 95% CI: 0.0001 to 0.0023). A reverse indirect effect from FI-IRT to later SRS via SII was likewise observed (*B* = 0.0049, 95% CI: 0.0013 to 0.0110). Taken together, these findings underscore the interrelated longitudinal associations among sarcopenia risk, social isolation, and frailty. Social isolation may represent a longitudinal pathway linking earlier sarcopenia risk to subsequent frailty. Individual differences also appear to play a crucial role in shaping these longitudinal associations.

### 3.5 Subgroup and sensitivity analyses

Additional analyses were conducted to evaluate the robustness of the main findings. Multi-group RI-CLPMs showed no evidence of significant subgroup differences across age, sex, educational attainment, or residence. Detailed model fit comparisons and subgroup-specific estimates are reported in Supplementary Tables S10–S14.

Sensitivity analyses using alternative frailty measures, alternative missing-data handling, and sample restriction yielded findings that were broadly consistent with the primary RI-CLPM. Specifically, the main model was re-estimated using FI-DA, the *LL* transformed frailty measure, MI, and a restricted sample excluding participants classified as frail at baseline. Across these specifications, the main longitudinal associations involving sarcopenia risk were generally preserved (Supplementary Table S15).

The reverse pathway from frailty to later sarcopenia risk through social isolation was the most robust pattern. The component paths from *T*_1_ frailty to *T*_2_ social isolation and from *T*_2_ social isolation to *T*_3_ sarcopenia risk remained evident across alternative models. Bootstrap-based indirect-effect analyses showed a similar pattern, with stronger and more stable support for the reverse indirect pathway than for the forward pathway (Supplementary Table S16). The forward pathway from sarcopenia risk to later frailty through social isolation was directionally retained across sensitivity analyses, although statistical support for the path from *T*_2_ social isolation to *T*_3_ frailty was attenuated under the log-logistic specification. In addition, the autoregressive effects of sarcopenia risk remained non-significant in the alternative frailty-measure models and in the model excluding baseline frailty, but became significant in the MI model. Overall, these analyses supported the robustness of the primary findings while indicating that the reverse indirect pathway was more consistent than the forward pathway.

## 4 Discussion

Using three waves of CHARLS data and random-intercept cross-lagged panel models, we addressed two research questions and their corresponding hypotheses. First, we examined whether sarcopenia risk and frailty were bidirectionally associated at the within-person level. The results supported this hypothesis. Higher-than-usual sarcopenia risk predicted greater subsequent frailty, while higher-than-usual frailty predicted greater subsequent sarcopenia risk. Second, we tested whether social isolation acted as a within-person longitudinal pathway between sarcopenia risk and frailty. This hypothesis was also supported, although the indirect effects were modest and evidence was more consistent for the reverse pathway. These patterns were broadly consistent across age, sex, education, and residence. Together, these findings suggest that sarcopenia risk, social isolation, and frailty may unfold as a linked within-person longitudinal process. This integrated, within-person perspective may help inform more coordinated and better-timed approaches to healthy ageing.

The interrelationships observed in the present study may be understood within behavioral, social, and geriatric frameworks. At the within-person level, the findings suggest that social isolation may represent a longitudinal indirect pathway linking sarcopenia risk and frailty. This pattern indicates that the pathway from early muscle-related vulnerability to later frailty is not purely physical in nature, but may be transmitted, at least in part, through the erosion of social connectedness. This interpretation is consistent with prior evidence that social isolation and loneliness are associated with sarcopenia among middle-aged and older adults in China, as well as with CHARLS-based longitudinal evidence that loneliness and frailty are interrelated over time (48, 49). It is also supported by broader evidence linking frailty with social vulnerability, although the direction and strength of these associations appear to vary across measurement approaches, study populations, and follow-up intervals (14).

### 4.1 Forward pathway from sarcopenia risk to frailty

The forward pathway suggests that increases in sarcopenia risk may contribute to later frailty both directly and indirectly through social isolation. A plausible explanation is that increasing sarcopenia risk may restrict mobility, reduce confidence in outdoor activity, and narrow opportunities for social participation. Over time, these changes may shrink an older adult’s social world and increase the likelihood of withdrawal from interpersonal contact and community engagement. In turn, reduced participation may reinforce inactivity, deconditioning, and declining physiological reserve, thereby accelerating frailty progression. This interpretation is also in line with longitudinal evidence showing that higher social participation is associated with lower frailty, whereas loneliness is associated with higher frailty levels in older adults (50). It is also consistent with prior evidence showing that frailty, quality of life, and loneliness are closely intertwined among older Chinese adults (51).

Several mechanisms may help explain why social isolation occupies such a central position in this process. First, social isolation is likely to reduce opportunities for daily movement, structured activity, and role fulfillment, all of which are important for maintaining muscle function and resilience in later life. Second, socially isolated older adults may have less access to emotional support, instrumental assistance, and health-related feedback, which may delay the recognition and management of early functional decline. Third, prolonged social disconnection may contribute to poorer health behaviors, including lower adherence to exercise, nutritional management, and self-care. Collectively, these pathways may amplify the impact of sarcopenia risk on subsequent frailty and help explain why the mediating role of social isolation was evident at the within-person level. This interpretation is also in line with prior work suggesting that muscle health should be regarded as an integral component of healthy aging and that early identification of muscle loss in routine practice is essential for preventing downstream functional decline (52). This view is further supported by recent longitudinal evidence indicating that worsening sarcopenia status is associated with greater subsequent frailty risk and faster frailty progression in Chinese older adults (53).

This interpretation should not be understood as reflecting simple measurement overlap between sarcopenia risk and frailty. Although the SRS and FI-IRT may be partly linked through physical function, their modest between-person association indicates that they are not interchangeable measures of the same construct. Instead, the SRS appears to reflect muscle-related vulnerability, whereas the FI-IRT reflects broader multidomain deficit accumulation. This distinction is important when interpreting the cross-lagged findings, because the observed associations should be understood as links between related but non-identical domains of ageing-related vulnerability.

The within-person cross-lagged effects were statistically significant but modest in magnitude. The significant standardized coefficients ranged from approximately 0.04 to 0.07. When interpreted against empirical benchmarks for cross-lagged models (54), these estimates correspond to small-to-moderate within-person effects, as values near 0.03, 0.07, and 0.12 have been proposed to represent small, medium, and large cross-lagged effects, respectively (55). Their practical meaning depends on the scale of inference. At the individual level, estimates of this size should be interpreted cautiously, as they are unlikely to provide sufficient precision for clinical prediction on their own. At the population level, however, modest within-person effects may still be meaningful in this context. Social isolation, sarcopenia, and frailty are common age-related conditions that can co-occur and evolve over time in older adults (9, 13, 19). These findings carry particular weight from a population-health perspective, complementing their clinical implications. They may help identify potential prevention targets and inform resource allocation for healthy ageing initiatives.

At the same time, the attenuation of the forward pathway under the *LL* frailty-score specification should be interpreted in relation to the frailty metric, rather than as evidence of a general weakening of the forward temporal pattern. One plausible explanation is that *LL*-transformed scores were more concentrated at the lower end of the distribution (44). This metric may therefore provide less local measurement information in the low-to-moderate frailty range and may be less sensitive to subtle within-person changes. More broadly, monotonic transformations of an IRT latent scale can preserve rank ordering while changing the score metric and the distribution of test information. These changes may affect the detection of small longitudinal effects (56). In the present analyses, the attenuation was limited to the social isolation-to-frailty component of the forward pathway. Under the *LL* specification, the within-person cross-lagged paths from sarcopenia risk to subsequent social isolation and frailty remained positive and statistically supported. Thus, the *LL* result mainly suggests that the estimated magnitude and precision of the social-isolation-mediated pathway should be interpreted with caution, whereas the broader interpretation of sarcopenia risk as an early signal of physical vulnerability remains supported by the overall pattern of findings.

### 4.2 Reverse pathway in the broader literature

The present study also extends previous work by clarifying the reverse sequence from frailty to later sarcopenia risk through social isolation. Prior longitudinal studies provide partial evidence for these links (10, 13, 57, 58). However, few have examined sarcopenia risk, social isolation, and frailty within the same longitudinal framework. Their ordering and directionality therefore remain unclear. Recent RI-CLPM research has documented bidirectional associations between social relationships and frailty in older adults, but sarcopenia risk has rarely been examined together with social isolation and frailty within the same within-person longitudinal framework (19).

Frailty predicted later social isolation. Social isolation, in turn, predicted later sarcopenia risk. These findings suggest that the three conditions do not follow a simple one-way pathway. Instead, they may form a reciprocal and self-reinforcing cycle over time. The stronger support for the reverse indirect pathway may reflect the more immediate impact of established frailty on social functioning and social participation. Frailty is a multidomain state of reduced physiological reserve. Once it becomes clinically apparent, fatigue, weakness, slower gait, and functional limitation may quickly restrict mobility, reduce confidence in outdoor activity, and narrow social roles. These changes may lead to social withdrawal and greater dependence on others. By contrast, early sarcopenia risk may initially reflect subtler changes in muscle-related function. Its effect on social isolation may therefore be more gradual and less immediately visible. In turn, greater social isolation may promote sedentary behaviour and reduce opportunities to maintain muscle strength and physical function. Over time, this may contribute to a higher risk of sarcopenia.

Few studies have directly tested this full reverse pathway, but recent evidence supports its component links. RI-CLPM studies suggest that frailty may precede later social isolation and loneliness in older adults (19, 59), and longitudinal evidence indicates that social isolation predicts later possible sarcopenia risk (13). Nevertheless, prior findings on the frailty–social connection relationship are not entirely consistent. A systematic review reported overall associations between frailty and multiple dimensions of social vulnerability, but highlighted substantial heterogeneity in measurement, which may limit comparability across studies and partly account for differences in findings (14). Further cohort and intensive longitudinal evidence suggests that these associations may vary according to the social construct assessed, exposure duration, follow-up interval, and whether within-person change is distinguished from between-person differences (60, 61). Thus, our findings build on prior component-level evidence by demonstrating how frailty, social isolation, and sarcopenia risk are linked within a single within-person longitudinal framework.

From a clinical perspective, interventions targeting different points in this cycle may have broader downstream benefits. This reverse pathway is also consistent with a social frailty perspective informed by Social Production Function theory (62). From this perspective, declines in physical reserve and unmet social needs may become mutually reinforcing in later life. Notably, the bootstrap analyses provided more consistent support for this reverse indirect pathway than for the forward pathway, suggesting that frailty-related social withdrawal may be a particularly stable route through which later sarcopenia risk is reinforced.

### 4.3 Clinical and public health implications

These findings have several clinical and public health implications. Sarcopenia risk may represent an upstream and modifiable intervention target, whereas social isolation may be an important pathway in the progression to frailty. The practical implication is not that sarcopenia risk, social isolation, and frailty should be addressed as separate problems. Rather, our findings support an integrated prevention approach in which muscle-related vulnerability, social disconnection, and early frailty are assessed and addressed together in older adults.

First, primary care and community health services should prioritize early identification of older adults with elevated sarcopenia risk before frailty is firmly established. The sarcopenia risk score used in this study was derived from a small set of non-invasive indicators, including estimated skeletal muscle mass, grip strength, and gait speed. All can be obtained from routine anthropometric and functional assessments. This makes the measure simple, low-cost, repeatable, and feasible for use in primary care and community settings. This approach may be especially useful for older adults in real-world, resource-constrained settings, where low-burden screening tools are needed and reference assessments such as dual-energy X-ray absorptiometry are often difficult to obtain. Because the score is continuous rather than dichotomous, it preserves the full gradient of risk and can be repeated during routine visits. This may support repeated monitoring of muscle-related vulnerability before frailty becomes established. Recent evidence also supports the use of simple sarcopenia screening approaches in older adults, especially in settings where feasibility, repeatability, and low implementation burden are essential (63, 64).

Second, assessment of social isolation should be integrated into sarcopenia-risk and frailty screening. Prevention should not focus on muscle decline alone, but should also address concurrent losses in social connectedness. For older adults with weak grip strength, slower gait speed, early functional limitation, or pre-frailty, clinicians and community health workers should also ask about social participation, living arrangement, and contact with children, relatives, friends, or neighbors. This combined screening may help identify older adults who are both physically vulnerable and socially disconnected, a group likely to benefit from timely social and community support (48).

Third, prevention strategies could be informed by the observed direction of risk. For older adults with elevated sarcopenia risk but without established frailty, the immediate priority is to preserve muscle function while maintaining social participation. Practical strategies may include resistance and balance exercise, adequate protein and energy intake, and referral to accessible group-based or community activities. These components could be embedded in home- or community-based programmes. For example, existing evidence suggests that strength-oriented models incorporating *Tai Chi* elements may be feasible in non-specialist settings (65). For older adults who are already pre-frail or frail, the stronger reverse pathway observed in this study points to a complementary priority: preventing social withdrawal. Fatigue, weakness, slow gait, and functional limitation may reduce confidence in outdoor activity and narrow social roles. Frailty management should therefore combine exercise and nutritional support with practical support for maintaining mobility, family contact, peer interaction, and participation in community life (12, 66–70). When social disconnection is identified, follow-up actions may include referral to exercise groups, nutrition counselling, senior-center activities, volunteer or neighborhood-support programmes, and digital communication training (12, 68–71). Tailored digital-skills support may be useful when it helps older adults maintain regular contact, access health information, and remain engaged in community life. Community-level responses may also incorporate social prescribing pathways that connect high-risk older adults to non-clinical resources (12, 68–70). These actions are practical because they can be delivered through existing primary care, public health, and community ageing-service platforms, rather than requiring highly specialized clinical infrastructure. Where feasible, they should be embedded within accessible and age-friendly community settings, with attention to transportation, barrier-free activity spaces, communication support, digital inclusion, and both online and offline social participation (68–70, 72).

Finally, these recommendations should be viewed as actionable priorities for risk stratification and integrated prevention. The longitudinal design and RI-CLPM framework strengthen the temporal interpretation of our findings. However, given the modest RI-CLPM effect sizes observed in this study, intervention studies are needed to determine whether these temporally ordered associations are modifiable and clinically meaningful. Our findings suggest that prevention should focus on older adults at early risk. Integrated strategies should address muscle function, nutritional support, mobility maintenance, and social connectedness (12, 48, 67, 71, 73, 74). Future pragmatic trials and implementation studies should test whether these strategies can be delivered effectively in primary care and community settings. They should also examine whether these strategies improve risk stratification, produce clinically meaningful changes, slow frailty progression, or reduce subsequent sarcopenia risk.

### 4.4 Sociocultural context and generalizability

These findings should also be interpreted within China’s distinctive sociocultural context. In Chinese society, family-based support and intergenerational ties remain central to late-life care. They are reinforced by enduring norms of filial piety and form a core component of older adults’ social networks (75, 76). This family-centred context may help explain why social isolation featured so prominently in the observed pathways. A weakening of intergenerational connectedness may carry particularly strong implications for physical function and frailty.

This context also shapes how our social isolation measure should be interpreted. The intergenerational dimension of our index captured the frequency of face-to-face and remote contact with non-cohabiting children. However, it did not capture residential proximity between older adults and their children. Geographic proximity is a distinct structural feature of intergenerational relationships in China. Children who live nearby may be perceived as more filial, whereas more distant children may compensate through financial transfers and frequent remote contact (76). The geographic distribution of adult children has also been linked to depressive-symptom trajectories among older parents in rural China (77). Because contact frequency and physical proximity are only partly aligned, this unmeasured structural feature may have shaped the associations reported here in ways that our index could not capture.

Finally, China’s family-centred model of later-life care may qualify the generalizability of our findings. The importance of family ties for well-being is culturally patterned: in more collectivistic societies, the absence of family interaction appears to be more strongly associated with loneliness than in more individualistic societies (78). In China, family relationships remain a central source of social embeddedness and support in later life. Accordingly, the pathways linking social isolation to sarcopenia risk and frailty may be particularly salient in this setting. Our findings may therefore be most directly applicable to China and other family-centred ageing contexts. Replication in other cultural settings is needed to determine the extent to which these associations generalize beyond this context.

### 4.5 Strengths and limitations

This study has several important strengths. First, we used three waves of nationally representative CHARLS data and focused on adults aged 60 years and older, which enhances the public health relevance of the findings. Second, we examined sarcopenia risk, social isolation, and frailty within a single longitudinal framework, allowing a more integrated assessment of their longitudinal bidirectional associations than pairwise analyses. Third, we applied the RI-CLPM to distinguish stable between-person differences from within-person change over time. This distinction is important because prevention and intervention primarily target intra-individual change, rather than simply identifying who is at higher overall risk. Fourth, frailty was operationalized using an IRT-derived index rather than a conventional equal-weight deficit count. This represents a measurement strength because conventional cumulative-deficit frailty indices generally assume that all deficits contribute equally to frailty, despite differences in their clinical relevance and psychometric informativeness (79–81). By estimating item-specific discrimination and threshold parameters, the GRM allowed deficits to contribute differentially to the latent frailty score. This approach may improve score precision at the individual level and better represent heterogeneity in deficit severity and informativeness. Fifth, the sarcopenia risk score was derived from three non-invasive indicators that can be obtained in routine examinations and basic functional assessments, making it simple, low-cost, and suitable for repeated use in primary care and community settings. Finally, the main findings were supported by both sensitivity and multigroup analyses. The associations remained broadly consistent across alternative frailty specifications, and no significant subgroup differences were observed across age, sex, educational attainment, or residence.

Several limitations should also be acknowledged. First, although the indicators used to derive sarcopenia risk, social isolation, and frailty have been widely applied in Chinese population studies, they remain subject to measurement error and reporting bias. Second, follow-up attrition and extreme-value screening may have reduced sample representativeness. Both processes may have retained a healthier cohort with a narrower risk distribution. However, the attrition analysis showed no substantial baseline imbalance between included and excluded participants. The observed differences were mainly age-related, as expected in longitudinal ageing studies. The influence of extreme-value exclusion is also likely to be limited. We applied a conservative three-interquartile-range criterion, and the exclusion rate was modest. The same rule was re-applied in the multiple-imputation sensitivity analysis, and the main pattern of findings was preserved. Therefore, healthy-survivor bias and range restriction cannot be fully ruled out. Nevertheless, they are unlikely to materially alter the interpretation of the main longitudinal findings. If present, these biases would more likely attenuate, rather than inflate, the within-person associations. Third, the 4-year observation window and relatively long intervals between waves may have limited the detection of short-term fluctuations and transitions. From a measurement perspective, although the graded response model was conceptually appropriate for the ordered categorical frailty items, the 40-item frailty index spans multiple health domains, and residual multidimensionality may have weakened model fit and parameter robustness. Future studies should therefore evaluate multidimensional IRT approaches to better capture the multidomain structure of frailty. Additionally, the social isolation index assessed structural isolation rather than subjective loneliness, and these constructs may operate through partly distinct mechanisms. Moreover, reduced mobility, lower social participation, and poor nutritional status may partly explain the observed associations. These explanations are indirectly supported by prior literature but were not tested as mediating processes in this study. This was mainly because relevant mediators were not consistently measured across waves or could overlap with items in the multidomain frailty construct, making their role as independent mediators difficult to interpret. Further studies with repeated mediator assessments are needed to clarify these pathways over time. Finally, although the RI-CLPM strengthened the temporal interpretation of the findings by distinguishing within-person dynamics from stable between-person differences, the study remains observational. RI-CLPM does not remove unmeasured time-varying confounding, and the bootstrap-based indirect effects should therefore be interpreted as modest temporal associations rather than as evidence of causal mediation (82, 83). In addition, some adjusted time-varying covariates, such as smoking and drinking, may partly reflect earlier changes in social connectedness or physical vulnerability rather than functioning solely as exogenous confounders. Future studies should use denser repeated assessments, objective measures of muscle mass and function, and inflammatory biomarkers. These data would help distinguish confounding, mediation, and downstream behavioral change more clearly. Dedicated prediction studies are also needed to quantify absolute risk differences, minimal important differences, and incremental predictive value. In parallel, pragmatic intervention trials should evaluate intervention-relevant thresholds and determine whether these temporally ordered associations can improve risk stratification and intervention targeting.

## 5 Conclusion

This study provides new longitudinal evidence that social isolation may be an important pathway linking early sarcopenia risk to later frailty severity in older adults. Rather than serving as a peripheral social correlate, social isolation may be embedded in the process through which early muscle-related vulnerability evolves into broader frailty accumulation. Moreover, the bootstrap-based indirect-effect findings further support this interpretation and suggest that the reverse pathway from frailty to later sarcopenia risk via social isolation may be more robustly maintained. Taken together, these findings indicate that sarcopenia risk, social isolation, and frailty may form a reciprocal and self-reinforcing process over time. They further underscore the importance of incorporating social health into frailty prevention frameworks. Accordingly, earlier identification of older adults with elevated sarcopenia risk and emerging social disengagement may offer a valuable opportunity to delay or attenuate frailty progression.

## Data Availability

The original contributions presented in the study are included in the article/Supplementary material, further inquiries can be directed to the corresponding authors.

## Footnotes

1
http://charls.pku.edu.cn/
