# Supplementary material for: Longitudinal bidirectional associations among sarcopenia risk, social isolation, and frailty in Chinese older adults using an item response theory-derived frailty index
Source: Front Public Health. 2026 Jul 8;14:1864444. doi: 10.3389/fpubh.2026.1864444 (PMC13393455; doi:10.3389/fpubh.2026.1864444)
Supplement: Supplementary file 1 [file Supplementary_file_1.pdf]

Supplementary Material

Longitudinal Bidirectional Associations Among Sarcopenia Risk, Social Isolation, and Frailty in Chinese Older Adults Using an Item Response Theory-Derived Frailty Index

Hui-Qian Lin[1,2#], Ci Lin[2#], Ya-Fen Lin[2], Xiao-Di Wang[2], Han-Bing Lin[2], Xin-Ying Chen[2], Wu-Xuan Huang[2], Jin-Lan Zhong[1], Hong Fan[1*], Ya-Jun Hong[1*]

[1] Zhongshan Hospital, Fudan University (Xiamen Branch), Fujian Province, 361006, China

[2] State Key Laboratory of Vaccines for Infectious Diseases, Xiang An Biomedicine Laboratory, Xiamen, 361102, China

SUPPLEMENTARY METHODS

Methods S1. Participant Selection.
Methods S2. Attrition Analysis.
Methods S3. Derivation of the Sarcopenia Risk Score.
Methods S4. Construction of the Social Isolation Index.
Methods S5. Derivation and Psychometric Evaluation, and Longitudinal Measurement Invariance of the Item Response Theory–Derived Frailty Index.
Methods S6. Covariate Definitions and Coding.
Methods S7. Sensitivity Analyses.
Methods S8. Statistical Software and R Packages.

SUPPLEMENTARY TABLES

Table S1. Item-level attrition comparison among participants with available raw 2011 baseline item-level data.
Table S2. Demographic attrition comparison within the broader baseline pool of main respondents aged 60 years or older.
Table S3. Items and Scoring of the 40-Item Frailty Index in the China Health and Retirement Longitudinal Study.
Table S4. Item Response Theory Assumption Tests and Graded Response Model Fit for the Frailty Index.
Table S5. Graded Response Model Item Parameters and Fit Statistics Across Waves.
Table S6. Fit Indices and RMSD-Based DIF Diagnostics for Longitudinal Measurement Invariance of the GRM-Based FI-IRT.
Table S7. RMSD-Based DIF Diagnostics and Likelihood-Ratio DIF Results for All 40 FI-IRT Items Across Three Waves.
Table S8. Spearman Correlation Coefficients and Distributions of Study Variables.
Table S9. Standardized Path Coefficients for the Four Best-Fitting Panel Models.

Table S10. Model Fit and Comparisons for Multigroup Random-Intercept Cross-Lagged Panel Models.
Table S11. Multigroup Random-Intercept Cross-Lagged Panel Model Results by Age, CHARLS, 2011–2015.
Table S12. Multigroup Random-Intercept Cross-Lagged Panel Model Results by Sex, CHARLS, 2011–2015.
Table S13. Multigroup Random-Intercept Cross-Lagged Panel Model Results by Educational Attainment, CHARLS, 2011–2015.
Table S14. Multigroup Random-Intercept Cross-Lagged Panel Model Results by Residence, CHARLS, 2011–2015.
Table S15. Sensitivity Analyses of Cross-Lagged Path Estimates Across Alternative Model Specifications.
Table S16. Bootstrap-based tests of longitudinal indirect effects in the primary and sensitivity RI-CLPMs (unstandardized estimates).

SUPPLEMENTARY FIGURES

Figure S1. Participant Selection Flowchart.
Figure S2. Scree Plots From Principal Component Analysis of the Polychoric Correlation Matrix Across Waves.
Figure S3. Test Information and Standard Error Curves for the 40-Item Frailty Index Across Waves.
Figure S4. Test Characteristic Curve–Based Anchoring of the Frailty Threshold and Distribution of Baseline Latent Frailty Scores.
Figure S5. Distributions and Concordance of Alternative Frailty Measures Across Waves.

SUPPLEMENTARY METHODS

Methods S1. Participant Selection.

Data were obtained from Waves 1 to 3 (2011, 2013, and 2015) of the China Health and Retirement Longitudinal Study (CHARLS). The 2011 baseline survey included 17,705 participants. Participant selection proceeded sequentially to establish the longitudinal analytic cohort for the present study.

At Wave 1, participants were excluded if they were non-main respondents (*n* = 1,991), were younger than 60 years (n = 8,950), or had missing key study variables at baseline (*n* = 1,185). For the sarcopenia risk score (SRS) and social isolation index (SII), a wave-specific score was considered missing when all component variables required to construct the corresponding measure were missing at that wave. For the frailty index (FI), a wave-specific score was considered missing when more than 20% of frailty index constituent deficits were missing (1). After these baseline exclusions, 5,579 participants remained eligible.

Among these 5,579 eligible participants, those with missing SRS, SII, or FI at Wave 2 were excluded (*n* = 1,307), leaving 4,272 participants. Participants with missing SRS, SII, or FI at Wave 3 were then further excluded (*n* = 816), resulting in 3,456 participants with complete repeated measurements across Waves 1 to 3.

In addition, participants were required to have data available for at least one covariate. No participants were excluded at any wave due to complete missingness of all covariates.

After data cleaning, missing-data imputation, and derivation of the study variables, including the sarcopenia risk score (SRS), social isolation index (SII), and item response theory-derived frailty index (FI-IRT), extreme outlier screening was conducted for 9 wave-specific study variables. These variables included SRS, SII, and FI-IRT scores across the three waves. Extreme outliers were defined as values more than three interquartile ranges (IQRs) below the first quartile or above the third quartile. Participants with at least one extreme outlier at any wave were excluded (*n* = 297). The final analytic sample therefore comprised 3,159 participants. The detailed participant selection process is shown in Figure S1.

Methods S2. Attrition Analysis.

To evaluate the potential for selection bias due to attrition or missing data, we compared baseline characteristics between participants included in the final analytic sample and those excluded from the final longitudinal analysis. Because participants excluded for missing baseline study variables could not all be compared across the complete baseline item profile, the attrition analysis was presented in two parts.

First, among participants with available raw 2011 baseline item-level data, we compared the final analytic sample with participants who were not retained in the final longitudinal analytic cohort because of missing repeated measures, loss to follow-up, or data-processing exclusions. This item-level comparison included 3,159 retained participants and 2,470 excluded participants and is presented in Supplementary Table S1. Second, to include participants excluded because of missing baseline study variables, we compared demographic characteristics between the final analytic sample and all excluded participants within the broader baseline pool of CHARLS 2011 main respondents aged 60 years or older. This demographic comparison included 3,159 participants in the final analytic sample and 3,605 excluded participants and is presented in Supplementary Table S2.

All item-level comparisons were based on raw 2011 baseline items. The modeled FI-IRT, sarcopenia risk score, and social isolation index were not used in the attrition analysis because excluded participants were not processed through the full score-construction pipeline. Continuous and ordinally scored variables were summarized as means and standard deviations, and categorical variables as numbers and percentages. Percentages were calculated among participants with non-missing observed data for each raw baseline item.

Between-group differences were evaluated primarily using standardized differences, reported as standardized mean differences (SMDs), for consistency with baseline-comparison and balance-diagnostic literature (2,3). For continuous variables and scored item-level variables summarized as mean (SD), SMDs were calculated as standardized mean differences; for binary variables, they were calculated as standardized differences in proportions. This approach was used descriptively because the scored item-level variables were presented on their original numeric scales rather than modeled as latent constructs. Values of $|SMD| < 0.10$ were interpreted as negligible, 0.10–0.20 as small, and ≥ 0.20 as potentially meaningful imbalance. The 0.10 threshold follows commonly used covariate-balance conventions, whereas the graded interpretation of larger standardized differences was informed by conventional effect-size benchmarks (4,2,3). *P* values from chi-square tests for categorical variables and one-way analyses of variance for variables summarized as SD were reported for descriptive reference only and were not used as the primary basis for interpretation, because *P* values are strongly influenced by sample size and do not directly quantify practical imbalance (5,6).

The attrition analysis did not indicate broad or systematic imbalance across most baseline domains. Most chronic conditions, social-isolation components, health behaviors, and most depressive-symptom and cognitive items showed negligible or small standardized differences. Potentially meaningful differences were mainly observed for age and

physical-function indicators. Compared with excluded participants, retained participants were younger, had fewer baseline functional limitations, better grip strength, and a lower prevalence of figure-copying failure. In the broader demographic comparison including all excluded participants, rural residence also showed a potentially meaningful difference. Overall, these findings suggested selective but clinically interpretable attrition rather than diffuse imbalance across the baseline profile. Complete results are shown in Supplementary Tables S1 and S2.

Methods S3. Derivation of the Sarcopenia Risk Score.

Direct measurements of appendicular skeletal muscle mass (ASM) by dual-energy X-ray absorptiometry (DXA) or bioelectrical impedance analysis (BIA) were not available in the China Health and Retirement Longitudinal Study (CHARLS). Therefore, ASM was estimated using a validated predictive equation, with sex coded as 1 for men and 2 for women:

$$\boldsymbol{ASM = 0.193 \times weight\ (kg) + 0.107 \times height\ (cm) - 4.157 \times sex - 0.037 \times age\ (years) - 2.631} \quad \textbf{(1)}$$

The skeletal muscle mass index (SMI) was then calculated as:

$$\boldsymbol{SMI = \frac{ASM}{height^2\ (m)^2}} \quad \textbf{(2)}$$

Handgrip strength was defined as the maximum value obtained across four trials, with two measurements taken from each hand using a mechanical dynamometer. Physical performance was assessed as usual-pace walking speed. Participants completed two 2.5-meter walk tests, and the faster speed was retained for analysis.

The continuous sarcopenia risk score (SRS) was constructed from SMI, handgrip strength, and walking speed. Each component was standardized within sex-specific strata. The standardized values (z-scores) were then reverse-coded so that higher values consistently indicated greater sarcopenia risk. The final SRS was calculated as the arithmetic mean of the three reversed z-scores (7).

Methods S4. Construction of the Social Isolation Index.

Social isolation was assessed using the social isolation index (SII), adapted from the Berkman–Syme Social Network Index (8). The SII comprised four binary dimensions: marital status, social engagement, organizational participation, and intergenerational contact (9). Marital status was coded as 1 for unmarried participants and 0 for those who were married or partnered. Social engagement and organizational participation were each coded as 1 if no relevant activity had been undertaken during the past month and 0 otherwise. Intergenerational contact was defined by the frequency of face-to-face meetings and remote contact with non-cohabiting children. For participants with multiple non-cohabiting children, the highest frequency across all children was identified separately for each contact type, and the higher of the two values was used to represent overall intergenerational contact. Contact at least once per week was coded as 0, and less frequent contact was coded as 1. The four indicators were summed to yield a total score ranging from 0 to 4, with higher scores indicating greater social isolation. A valid score required non-missing data on at least three dimensions.

Methods S5. Derivation and Psychometric Evaluation, and Longitudinal Measurement Invariance of the Item Response Theory–Derived Frailty Index.

S5.1. Item Coding, Graded Response Model Estimation, and Score Derivation

Frailty was defined using 40 health deficit items derived from the China Health and Retirement Longitudinal Study, spanning six domains: physician-diagnosed chronic diseases (14 items), functional limitations (8 items), activities of daily living (ADL; 6 items), instrumental activities of daily living (IADL; 5 items), cognition (4 items), and self-rated measures (3 items). Item coding followed standard deficit-accumulation principles, with higher values consistently indicating greater frailty burden. Chronic disease and self-rated items were dichotomized. Functional limitation, ADL, and IADL items were coded on a four-category ordinal scale: 0, no difficulty; 1, some difficulty; 2, much difficulty; and 3, unable to perform the task. Cognition items were scored using their original or transformed deficit metrics, as detailed in Table S3. Participants with >20% missing deficit items were excluded before imputation.

To represent frailty as a latent continuum rather than an unweighted sum, a graded response model (GRM) was estimated separately at each wave (10). For item *j* with ordered response categories, the cumulative probability of endorsing category *k* or higher was modeled as

$$P(Y_{ij} \geq k|\theta_i) = \frac{1}{1 + \exp\left[-a_j\left(\theta_i - b_{jk}\right)\right]}, \quad k = 1, \ldots, m_j \tag{1}$$

where θ_i denotes the latent frailty trait for participant *i*, a_j is the item discrimination parameter, and b_{jk} is the threshold parameter for category *k* of item *j*. The probability of endorsing a specific response category was then derived as

$$P(Y_{ij} = k|\theta_i) = P(Y_{ij} \geq k|\theta_i) - P(Y_{ij} \geq k + 1|\theta_i) \tag{2}$$

After confirmation of the core IRT assumptions (see Methods S5.2), a unidimensional GRM was fitted separately at each wave. Participant-specific latent frailty scores were estimated using expected a posteriori (EAP) scoring. For interpretability, scores were rescaled to a 0–1 metric using min–max normalization:

$$FI_{IRT,\,i} = \frac{\theta_i - \min(\theta)}{\max(\theta) - \min(\theta)} \tag{3}$$

Higher values indicate greater frailty. This IRT-derived frailty index (FI-IRT) was used in all subsequent analyses.

S5.2. Evaluation of IRT Assumptions, Overall Model Fit, and Item Fit

We evaluated core IRT assumptions and limited-information model fit for the GRM used to derive the FI-IRT. Diagnostics were conducted separately at each wave (2011, 2013, and 2015; $n = 3{,}456$ per wave; 40 items). Polychoric correlation matrices were computed to examine item relationships, and the GRM was estimated separately at each wave.

Two assumptions were evaluated at each wave: unidimensionality and local independence. For unidimensionality, factorability was assessed using the Kaiser–Meyer–Olkin (KMO) measure and Bartlett's test of sphericity. Because

most items were ordinal, principal component analysis (PCA) was performed on the polychoric correlation matrix (11). A unidimensional approximation was considered adequate when $\lambda_1/\lambda_2 > 3.0$, the first component explained >20% of the total variance, and the scree plot showed a clear elbow (12). Local independence was evaluated using Yen's Q_3 residual correlations. Item pairs with $|Q_3| \leq 0.36$ were considered locally independent (13). Overall model fit was evaluated using the M2 statistic, root mean square error of approximation (RMSEA), comparative fit index (CFI), Tucker–Lewis index (TLI), and standardized root mean square residual (SRMSR) (14), and item fit was evaluated using the $S\text{–}\chi^2$ statistic with a Bonferroni-corrected threshold ($\alpha = 0.05/40$; $P < 0.00125$) (15).

Across waves, KMO values ranged from 0.892 to 0.924, and Bartlett's test was significant at all waves (all $P < 0.001$), supporting adequate factorability. The eigenvalue ratio λ_1/λ_2 exceeded 3.0 at each wave (3.868, 3.760, and 4.013), supporting a unidimensional approximation for the 40-item item pool (Table S4; see also Figure S2 for wave-specific scree plots). The local-dependence rate was 0.0% at all waves, indicating no appreciable residual dependence after conditioning on the latent frailty trait.

The GRM showed acceptable overall fit for the 40-item frailty index. Although M_2 was significant at all waves, this result was interpreted cautiously given its sensitivity to large samples (14). Approximate fit indices supported acceptable model fit (RMSEA = 0.037–0.039; SRMSR = 0.041–0.049; CFI = 0.882–0.921; TLI = 0.875–0.917), with modest improvement across waves (Table S4). Item-level misfit under the Bonferroni-adjusted $S\text{–}\chi^2$ criterion was limited (5.0%, 0.0%, and 2.5% in 2011, 2013, and 2015, respectively), further supporting the adequacy of the model for score derivation.

Parameter estimates were stable across waves, and discrimination varied substantially across domains. The discrimination parameter (a) ranged from 0.10 to 3.48 (pooled mean = 1.27). Highly discriminating items were concentrated in the ADL and IADL domains, whereas several chronic disease and cognitive items showed weaker discrimination (Table S5). Difficulty parameters (b) showed a complementary pattern, with several low-prevalence chronic disease items displaying high thresholds. Overall, these parameter patterns indicate unequal measurement contributions across deficits. Wave-specific test information curves are shown in Figure S3 and illustrate the range of the latent frailty continuum over which the 40-item FI-IRT provides the greatest measurement precision.

Taken together, the eigenvalue-ratio evidence, null local-dependence rates, and favorable fit indices supported the practical adequacy of the unidimensional GRM for deriving the FI-IRT, which was rescaled to a 0–1 metric and used in the primary RI-CLPM analyses.

S5.3. Longitudinal measurement invariance of the FI-IRT

To examine the cross-wave measurement comparability of the 40-item FI-IRT, we conducted an IRT-based longitudinal measurement invariance analysis. A multiple-group graded response model (GRM) was fitted, with survey wave (2011, 2013, and 2015) specified as the grouping variable. This approach follows the IRT-based

measurement-invariance framework, in which item parameters are evaluated across groups or measurement occasions when score comparability is required (16). Before model estimation, response categories for the same frailty item were harmonized across waves to ensure comparable threshold structures.

Three nested models were estimated. The configural model allowed item parameters to vary freely across waves. The metric invariance model constrained item discrimination parameters to equality across waves. The scalar invariance model further constrained both item discrimination and threshold parameters to equality across waves. Because likelihood-ratio tests can be sensitive to large samples, they were reported as supplementary evidence rather than used as the primary decision criterion. Longitudinal invariance was judged mainly by changes in limited-information fit indices for ordinal IRT models, including C2-based CFI and RMSEA (17,18). Metric or scalar invariance was considered supported when ΔCFI ≥ -0.010 and ΔRMSEA ≤ 0.015 (19). AIC and BIC were used as additional fit criteria.

When full scalar invariance was not supported, item-level non-invariance was evaluated using RMSD-based DIF diagnostics. RMSD is an IRT-based item-fit statistic that quantifies the discrepancy between empirical and model-implied item response functions, with larger values indicating poorer item fit (20,21). Following prior IRT item-fit and measurement-invariance studies, RMSD was defined as:

$$\mathbf{RMSD}_{i,g}=\sqrt{\sum_{c=0}^{C_i}\int\left[P_{i,c,g}^{(\mathrm{obs})}(\theta)-P_{i,c}^{(\mathrm{mod})}(\theta)\right]^2 f_g(\theta)\ d\theta} \qquad (1)$$

where $P_{i,c,g}^{\mathrm{obs}}(\theta)$ denotes the observed category response function for item i, category c, in group g (wave); $P_{i,c}^{\mathrm{mod}}(\theta)$ denotes the model-implied category response function under the fully constrained (invariant) model; $f_g(\theta)$ denotes the latent trait density for group g; and C_i is the highest response category for item i. In the present polytomous GRM analysis, this formulation was applied to item category response functions across waves. RMSD was used as an effect-size indicator of item-level non-invariance. Based on prior RMSD-based DIF and partial-invariance applications, RMSD ≥ 0.050 was prespecified to flag medium-or-larger item-level non-invariance (22,20,23,24). This threshold was used as the primary item-level criterion to avoid relying solely on likelihood-based statistical significance in a large sample. Parameters of flagged items were freed, whereas the remaining items were retained as anchors to form an RMSD-informed partial scalar invariance model.

The configural model showed acceptable fit across the three waves (CFI = 0.9373, TLI = 0.9339, RMSEA = 0.0227), supporting the same unidimensional frailty construct over time. The metric invariance model showed negligible deterioration relative to the configural model (CFI = 0.9366, TLI = 0.9354, RMSEA = 0.0225; ΔCFI = −0.0007; ΔRMSEA = −0.0002), supporting invariance of item discrimination parameters. Full scalar invariance was not supported according to the ΔCFI criterion (CFI = 0.9237, TLI = 0.9268, RMSEA = 0.0241; ΔCFI = −0.0136; ΔRMSEA = 0.0014).

Traditional likelihood-ratio DIF tests flagged 23 of the 40 items as significant after Benjamini–Hochberg correction, suggesting that significance-based DIF testing may be sensitive in this large-sample setting (6). Therefore, RMSD was used as the primary item-level effect-size criterion for substantive non-invariance. The RMSD-based DIF analysis identified four items with medium-or-larger non-invariance: kneeling (RMSD = 0.050), arthritis (RMSD = 0.057), orientation_score (RMSD = 0.060), and memory_deficit (RMSD = 0.284). The parameters of these four RMSD-flagged items were then freed in an RMSD-informed partial scalar invariance model, whereas the remaining 36 items were retained as equality-constrained anchors. The partial scalar model showed acceptable fit and negligible deterioration relative to the configural model (CFI = 0.9329, RMSEA = 0.0224; ΔCFI = −0.0044; ΔRMSEA = −0.0003), meeting the recommended criteria for measurement invariance ($|\Delta\text{CFI}| < 0.010$ and $\Delta\text{RMSEA} < 0.015$) (19). These results supported partial scalar invariance across waves. Detailed invariance model comparisons and item-level DIF diagnostics are presented in Tables S6 and S7.

Overall, the FI-IRT captured a broadly comparable latent frailty construct across the 2011, 2013, and 2015 waves. Although full scalar invariance was not established, configural invariance, metric invariance, and RMSD-informed partial scalar invariance were supported, with only four items showing medium-or-larger item-level non-invariance. These findings support the longitudinal use of the FI-IRT in the RI-CLPM analyses while keeping the measurement-invariance claim appropriately bounded.

Methods S6. Covariate Definitions and Coding.

Covariates comprised time-invariant sociodemographic variables and time-varying health-related variables. Baseline time-invariant sociodemographic covariates included age, sex, educational attainment, and residence. Age was categorized as 60–74 years or ≥75 years. Sex was coded as male or female. Educational attainment was dichotomized as below high school versus high school or above. Specifically, the below-high-school category included no formal education, incomplete primary school with literacy, sishu/home school, elementary school, and middle school. The high-school-or-above category included high school, vocational school, two- or three-year college, four-year college, and postgraduate education. Residence was classified as urban or rural.

Time-varying health-related covariates assessed at each wave included depressive symptoms, drinking status, smoking status, and sleep quality. Depressive symptoms were measured using the 10-item Center for Epidemiologic Studies Depression Scale (CES-D-10) and treated as a continuous total score, with higher scores indicating greater symptom severity. Drinking status was coded as yes or no based on self-reported alcohol consumption at each wave. Smoking status was coded as yes or no based on self-reported smoking status at each wave. Sleep quality was categorized as good or poor. Good sleep quality was defined as a self-reported nightly sleep duration of 7–9 hours and a daytime nap duration of ≤60 minutes. All other patterns were classified as poor sleep quality.

Methods S7. Sensitivity Analyses.

In addition to the primary analysis, we conducted a series of sensitivity analyses to assess the robustness of the RI-CLPM findings to alternative frailty measures, missing-data handling, and sample restrictions. Detailed parameter estimates are provided in Table S11. All sensitivity models retained the same RI-CLPM structure as the primary analysis and included the same time-invariant covariates (age, sex, educational attainment, and residence) and time-varying covariates (depressive symptoms, smoking status, drinking status, and sleep quality).

First, we considered two alternative frailty measures. One was a traditional deficit-accumulation frailty index (FI-DA) constructed from the same 40 health deficits, each coded on a 0–1 scale and averaged across non-missing items. For participant i at wave w, this index was defined as

$$FI_{iw}^{\mathrm{DA}} = \frac{1}{K_{iw}} \sum_{j=1}^{K_{iw}} d_{ijw} \quad (1)$$

where d_{ijw} denotes the deficit score for item j, coded on a 0–1 scale, and K_{iw} is the number of non-missing deficits available for participant i at wave w. Detailed coding of the FI-DA components is shown in Table S3. The other was an log-logistic (*LL*)-transformed frailty measure derived from the same GRM-derived latent frailty trait. Let θ_{iw} denote the GRM latent frailty score for participant i at wave w. The GRM-derived frailty score was first linearly rescaled to the 0–1 range using the pooled minimum and maximum latent scores across Waves 1–3:

$$FI_{iw,01}^{\mathrm{GRM}} = \frac{\theta_{iw} - \theta_{\min}}{\theta_{\max} - \theta_{\min}} \quad (2)$$

The *LL*-transformed frailty score was then obtained as

$$FI_{iw,01}^{\mathrm{LL}} = \frac{\exp(\theta_{iw}) - \exp(\theta_{\min})}{\exp(\theta_{\max}) - \exp(\theta_{\min})} \quad (3)$$

This comparison was informed by prior methodological work showing that item response theory can be used to identify interpretable thresholds on the latent trait and to link them back to expected frailty index scores (25,26). Because frailty indices are bounded, right-skewed, and typically clustered at the lower end of the scale, we compared the empirical distributions of FI-DA, GRM-derived frailty scores, and *LL*-transformed frailty scores. The *LL*-transformed scores better captured the distributional characteristics of FI-DA, particularly its right-skewness and lower-end clustering, while preserving the same GRM-derived latent frailty construct. This distributional comparison is presented in Figure S5.

Second, to define baseline frailty for the sample-restriction sensitivity analysis, we used an internal test characteristic curve (TCC)-based anchoring approach to link the GRM-derived latent frailty trait to the FI-DA cut-

point of 0.25, a commonly used threshold in deficit-accumulation frailty research (27,28). Because the purpose of this sensitivity analysis was to exclude participants who were frail at baseline, the TCC was estimated from Wave 1 data only. This approach was conceptually related to the IRT thresholding frameworks described in prior studies (25,26), but differed in that the latent threshold was internally anchored to a conventional FI value rather than derived from an external clinical anchor item. At baseline (Wave 1), a one-factor graded response model was fitted. FI items with no response variation were excluded. Item category response probabilities were then estimated from the fitted model over a dense θ grid ranging from −4 to 4 in increments of 0.001.

For each theta value, the expected item-level deficit contribution was calculated by weighting category scores by their corresponding response probabilities and standardizing by the maximum possible category score for that item. The expected frailty index at each theta value was then obtained by averaging these standardized expected deficit contributions across all retained items:

$$E(\mathbf{FI}|\theta) = \frac{1}{J}\sum_{j=1}^{J} E\left(d_j|\theta\right) \quad (4)$$

where J is the number of retained FI items in the TCC analysis. The frailty threshold θ^* was defined as the theta value at which the expected frailty index was closest to 0.25:

$$\theta^* = \underset{\theta}{\mathbf{argmin}}|E(\mathbf{FI}|\theta) - \mathbf{0.25}| \quad (5)$$

This procedure yielded a threshold of θ^*= 1.341, corresponding to $E\left(FI|\theta^*\right)$= 0.24998. After linear rescaling of the GRM latent scores to the 0–1 range, this threshold corresponded to FI-IRT = 0.7038. Participants with baseline FI-IRT values at or above this threshold were classified as frail and excluded from the baseline non-frail sensitivity analysis. The distribution of baseline GRM-derived latent frailty scores and the TCC-anchored frailty classification are shown in Figure S4. This resulted in the exclusion of 195 participants, yielding an analytic sample of 2,964.

Third, to assess sensitivity to the missing-data strategy, we performed multiple imputation using predictive mean matching as implemented in the *mice* package in R (29). Five imputed datasets were generated (m = 5, maxit = 5, seed = 14532). Within each imputed dataset, all derived study variables were recalculated, including SRS and SII, and the GRM was refitted at each wave to re-estimate FI-IRT scores. Extreme outliers were defined as values more than three IQRs below the first quartile or above the third quartile. After outlier exclusion, each imputed dataset included 3,069 participants. RI-CLPMs were then fitted separately within each imputed dataset using the same specification as in the primary analysis, and the parameter estimates were pooled using Rubin's rules.

Methods S8. Statistical Software and R Packages.

All analyses were conducted in R version 4.4.1 (30). The *mirt* package was used for graded response model estimation and latent frailty score derivation, multiple-group GRM estimation, and IRT-based longitudinal measurement invariance and DIF analyses (31). The *psych* package was used to compute polychoric correlation matrices and related psychometric diagnostics (32). Primary missing-data imputation was performed using *missRanger* (33). Descriptive statistics, correlation analyses, and data management were conducted in base R and related packages. Longitudinal cross-lagged panel models, including CLPMs and RI-CLPMs, were fitted using *lavaan* (34). In sensitivity analyses, multiple imputation was implemented using *mice* (35).

SUPPLEMENTARY TABLES

Table S1. Item-level attrition comparison among participants with available raw 2011 baseline item-level data.

Characteristic	Included (*n* = 3,159)	Excluded (*n* = 2,470)	SMD	*P* value
No. of participants	3159	2470		
Demographics				
Age, years	67.24 (5.99)	68.81 (7.17)	−0.238[†]	< 0.001
Male	1641 (52.0)	1151 (46.6)	0.107	< 0.001
High school or above	545 (17.3)	415 (16.8)	0.012	0.684
Rural residence	1713 (84.6)	1207 (77.0)	0.194	< 0.001
***Chronic conditions, n* (%)**				
Hypertension	891 (28.3)	828 (33.7)	−0.117	< 0.001
Dyslipidemia	309 (9.9)	253 (10.4)	−0.017	0.559
Diabetes	180 (5.7)	202 (8.3)	−0.100	< 0.001
Cancer	26 (0.8)	17 (0.7)	0.015	0.676
Chronic lung disease	403 (12.8)	380 (15.4)	−0.077	0.005
Liver disease	120 (3.8)	110 (4.5)	−0.033	0.239
Heart disease	426 (13.5)	411 (16.7)	−0.088	0.001
Stroke	84 (2.7)	98 (4.0)	−0.073	0.007
Kidney disease	185 (5.9)	185 (7.5)	−0.066	0.016
Stomach/digestive disease	673 (21.3)	616 (25.0)	−0.086	0.001
Emotional/psychiatric problem	39 (1.2)	42 (1.7)	−0.039	0.179
Memory-related disease	55 (1.7)	66 (2.7)	−0.064	0.022
Arthritis	1139 (36.1)	1021 (41.4)	−0.108	< 0.001
Asthma	161 (5.1)	134 (5.4)	−0.015	0.623
Functional limitation items (score 0–3), mean (SD)				
Difficulty: Jogging 1 km	1.58 (1.40)	1.89 (1.36)	−0.227[†]	< 0.001
Difficulty: Walking 1 km	0.50 (0.96)	0.85 (1.21)	−0.322[†]	< 0.001
Difficulty: Rising from chair	0.33 (0.55)	0.49 (0.70)	−0.264[†]	< 0.001
Difficulty: Climbing several flights of stairs	0.75 (1.03)	1.06 (1.16)	−0.283[†]	< 0.001
Difficulty: Kneeling/crouching	0.50 (0.86)	0.71 (1.01)	−0.219[†]	< 0.001
Difficulty: Extending arms	0.17 (0.61)	0.33 (0.83)	−0.215[†]	< 0.001
Difficulty: Carrying weights	0.23 (0.71)	0.52 (1.04)	−0.323[†]	< 0.001
Difficulty: Picking up a coin	0.05 (0.33)	0.15 (0.60)	−0.205[†]	< 0.001
Difficulty: Dressing	0.05 (0.26)	0.13 (0.46)	−0.222[†]	< 0.001
Difficulty: Bathing	0.08 (0.35)	0.21 (0.62)	−0.268[†]	< 0.001
Difficulty: Eating	0.03 (0.20)	0.08 (0.38)	−0.178	< 0.001
Difficulty: Getting in/out of bed	0.05 (0.24)	0.13 (0.42)	−0.220[†]	< 0.001
Difficulty: Using the toilet	0.16 (0.47)	0.30 (0.65)	−0.250[†]	< 0.001

Difficulty: Controlling urination/defecation	0.06 (0.31)	0.13 (0.49)	−0.165	< 0.001
Difficulty: Doing household chores	0.14 (0.51)	0.34 (0.82)	−0.295†	< 0.001
Difficulty: Preparing hot meals	0.15 (0.57)	0.35 (0.85)	−0.271†	< 0.001
Difficulty: Shopping	0.14 (0.54)	0.40 (0.93)	−0.336†	< 0.001
Difficulty: Managing money	0.27 (0.78)	0.48 (1.01)	−0.225†	< 0.001
Difficulty: Taking medications	0.10 (0.42)	0.19 (0.61)	−0.174	< 0.001
Cognition				
Orientation (0–5), mean (SD)	0.43 (0.61)	0.49 (0.65)	−0.083	0.002
Serial-7 subtraction (0–5), mean (SD)	1.61 (1.81)	1.85 (1.87)	−0.132	< 0.001
Immediate word recall (0–10), mean (SD)	3.28 (1.88)	3.06 (2.04)	0.115	< 0.001
Delayed word recall (0–10), mean (SD)	2.39 (1.95)	2.20 (1.97)	0.097	< 0.001
Figure-copying failure, *n* (%)	1296 (42.0)	1236 (52.2)	−0.205†	< 0.001
***Self-rated status, n* (%)**				
Poor self-rated health	1740 (55.1)	1519 (61.5)	−0.131	< 0.001
Poor self-rated memory	2592 (83.1)	2035 (85.0)	−0.052	0.060
Low life satisfaction	354 (12.5)	331 (16.0)	−0.100	< 0.001
***Physical measurements* (raw)*, mean* (SD)**				
Height, cm	156.58 (9.70)	154.73 (16.66)	0.136	< 0.001
Weight, kg	56.55 (11.23)	55.89 (12.21)	0.056	0.037
Grip strength, trial 1 (kg)	26.37 (9.23)	24.56 (10.12)	0.186	< 0.001
Grip strength, trial 2 (kg)	27.90 (9.44)	25.95 (10.18)	0.198	< 0.001
Grip strength, trial 3 (kg)	27.81 (9.24)	25.78 (10.24)	0.209†	< 0.001
Grip strength, trial 4 (kg)	29.10 (9.55)	27.06 (9.95)	0.209†	< 0.001
Gait time, trial 1 (s)	4.57 (2.18)	5.33 (7.93)	−0.130	< 0.001
Gait time, trial 2 (s)	4.35 (2.04)	5.37 (19.14)	−0.075	0.004
***Social isolation components, n* (%)**				
Marital isolation	574 (18.2)	566 (22.9)	−0.118	< 0.001
Social-activity isolation	1630 (52.0)	1383 (57.5)	−0.110	< 0.001
Organizational isolation	2953 (94.2)	2227 (92.6)	0.067	0.015
Intergenerational isolation	387 (21.2)	333 (23.7)	−0.058	0.108
***Depressive symptom items (CES-D-10, score 0–3), mean* (SD)**				
CES-D-10 item 1	1.00 (1.10)	1.09 (1.16)	−0.075	0.006
CES-D-10 item 2	0.92 (1.08)	1.07 (1.15)	−0.135	< 0.001
CES-D-10 item 3	0.96 (1.05)	1.10 (1.12)	−0.122	< 0.001
CES-D-10 item 4	1.06 (1.14)	1.26 (1.22)	−0.171	< 0.001
CES-D-10 item 5 (reverse-scored)	1.29 (1.20)	1.40 (1.23)	−0.086	0.002
CES-D-10 item 6	0.31 (0.73)	0.43 (0.88)	−0.146	< 0.001
CES-D-10 item 7	1.04 (1.18)	1.18 (1.23)	−0.119	< 0.001
CES-D-10 item 8 (reverse-scored)	1.07 (1.12)	1.20 (1.17)	−0.120	< 0.001

CES-D-10 item 9	0.55 (0.96)	0.71 (1.07)	−0.156	< 0.001
CES-D-10 item 10	0.35 (0.78)	0.52 (0.93)	−0.204†	< 0.001
***Health behaviors, n* (%)**				
Smoking	1381 (43.7)	996 (40.3)	0.069	0.011
Drinking	987 (31.2)	708 (28.7)	0.056	0.039
Good sleep quality	1032 (32.9)	786 (32.8)	0.004	0.913

Note. This table compares retained and excluded participants with available raw 2011 baseline item-level data. Values are shown as mean (SD) or *n* (%). Percentages were calculated among participants with non-missing observed data for each item. Standardized differences are reported as SMDs; calculation and interpretation are described in Supplementary Methods S2. † indicates |SMD| ≥ 0.20. ADL = activities of daily living; CES-D-10 = 10-item Center for Epidemiologic Studies Depression Scale; FI-IRT = item response theory-derived frailty index; IADL = instrumental activities of daily living; SD = standard deviation; SMD = standardized difference.

Table S2. Demographic attrition comparison within the broader baseline pool of main respondents aged 60 years or older.

Characteristic	Included (*n* = 3,159)	Excluded (*n* = 3,605)	SMD	*P* value
No. of participants	3159	3605		
Age, years, mean (SD)	67.24 (5.99)	68.84 (7.36)	−0.238†	< 0.001
Male, *n* (%)	1641 (52.0)	1733 (48.1)	0.077	0.002
High school or above, *n* (%)	545 (17.3)	717 (19.9)	−0.069	0.005
Rural residence, *n* (%)	1713 (84.6)	1570 (72.1)	0.308†	< 0.001

Note. This table compares the final analytic sample with all excluded participants in the broader baseline pool. Values are shown as mean (SD) or *n* (%). Percentages were calculated among participants with non-missing observed data for each variable. Standardized differences are reported as SMDs; calculation and interpretation are described in Supplementary Methods S2. † indicates |SMD| ≥ 0.20. SD = standard deviation; SMD = standardized difference.

Table S3. Items and Scoring of the 40-Item Frailty Index in the China Health and Retirement Longitudinal Study.

Domain	Item	Description	GRM Scoring	FI-DA Scoring
Chronic diseases	1	Self-reported physician-diagnosed hypertension	Yes = 1, No = 0	Yes = 1, No = 0
	2	Self-reported physician-diagnosed dyslipidemia	Yes = 1, No = 0	Yes = 1, No = 0
	3	Self-reported physician-diagnosed diabetes or high blood sugar	Yes = 1, No = 0	Yes = 1, No = 0
	4	Self-reported physician-diagnosed cancer or malignant tumor	Yes = 1, No = 0	Yes = 1, No = 0
	5	Self-reported physician-diagnosed chronic lung disease	Yes = 1, No = 0	Yes = 1, No = 0
	6	Self-reported physician-diagnosed liver disease	Yes = 1, No = 0	Yes = 1, No = 0
	7	Self-reported physician-diagnosed heart disease	Yes = 1, No = 0	Yes = 1, No = 0
	8	Self-reported physician-diagnosed stroke	Yes = 1, No = 0	Yes = 1, No = 0
	9	Self-reported physician-diagnosed kidney disease	Yes = 1, No = 0	Yes = 1, No = 0
	10	Self-reported physician-diagnosed stomach or digestive disease	Yes = 1, No = 0	Yes = 1, No = 0
	11	Self-reported physician-diagnosed emotional, nervous, or psychiatric problems	Yes = 1, No = 0	Yes = 1, No = 0
	12	Self-reported physician-diagnosed memory-related disease	Yes = 1, No = 0	Yes = 1, No = 0
	13	Self-reported physician-diagnosed arthritis or rheumatism	Yes = 1, No = 0	Yes = 1, No = 0
	14	Self-reported physician-diagnosed asthma	Yes = 1, No = 0	Yes = 1, No = 0
Functional limitations	15	Difficulty with running or jogging about 1 *km*	0 = No, 1 = Some, 2 = Much, 3 = Cannot do	Any difficulty = 1, No difficulty = 0
	16	Difficulty with walking 1 *km*	Same as above	Same as above
	17	Difficulty with getting up from a chair after sitting for a long period	Same as above	Same as above
	18	Difficulty with climbing several flights of stairs without resting	Same as above	Same as above
	19	Difficulty with stooping, kneeling, or crouching	Same as above	Same as above
	20	Difficulty with reaching or extending arms above shoulder level	Same as above	Same as above
	21	Difficulty with lifting or carrying weights over 10 jin (≈ 5 *kg*)	Same as above	Same as above
	22	Difficulty with picking up a small coin from a table	Same as above	Same as above
ADL	23	Difficulty with dressing, including putting on shoes and socks	Same as above	Same as above
	24	Difficulty with bathing or showering	Same as above	Same as above
	25	Difficulty with eating, such as cutting up food	Same as above	Same as above
	26	Difficulty with getting in or out of bed	Same as above	Same as above
	27	Difficulty with using the toilet, including getting up and down	Same as above	Same as above
	28	Difficulty with controlling urination and defecation	Same as above	Same as above
IADL	29	Difficulty with doing household chores	Same as above	Same as above
	30	Difficulty with preparing hot meals	Same as above	Same as above
	31	Difficulty with shopping for groceries	Same as above	Same as above
	32	Difficulty with managing money, such as paying bills and keeping track of expenses	Same as above	Same as above
	33	Difficulty with taking medications	Same as above	Same as above

Domain	Item	Description	GRM Scoring	FI-DA Scoring
Cognition	34	Calculation deficit: number of errors in serial subtraction of 7 from 100 (five trials)	Ordered polytomous score from 0 to 5, defined as the number of incorrect responses; 0 = no error and 5 = all five responses incorrect	Errors/5 ≥ wave-specific median = 1; < median = 0
	35	Visuospatial deficit: failure to successfully redraw a previously shown figure	Failure = 1, Success = 0	Failure = 1, Success = 0
	36	Orientation deficit: number of errors in reporting the current date, month, day of week, year, and season	Ordered polytomous score from 0 to 5, defined as the number of incorrect responses; 0 = no error and 5 = all five responses incorrect	Errors/5 ≥ wave-specific median = 1; < median = 0
	37	Memory deficit: immediate word recall (0–10) + delayed word recall (0–10), total (0–20)	Total recall ≤ wave-specific median = 1; > median = 0	Total recall ≤ wave-specific median = 1; > median = 0
Self-rated measures	38	Self-rated health status	Poor/very poor = 1; Very good/good/fair = 0	Poor/very poor = 1; Otherwise = 0
	39	Self-rated memory	Poor/very poor = 1; Very good/good/fair = 0	Poor/very poor = 1; Otherwise = 0
	40	Life satisfaction	Not at all/not very satisfied = 1; Otherwise = 0	Not at all/not very satisfied = 1; Otherwise = 0

Note. CHARLS = China Health and Retirement Longitudinal Study; FI = Frailty Index; FI-IRT = Item Response Theory-derived Frailty Index; FI-DA = Deficit-accumulation Frailty Index; GRM = Graded Response Model; ADL = activities of daily living; IADL = instrumental activities of daily living.

Table S4. Item Response Theory Assumption Tests and Graded Response Model Fit for the Frailty Index.

Year	KMO	Bartlett's $\chi^2(df)$	λ_1/λ_2	LD (%)	$M_2(df)$	RMSEA	CFI	TLI	SRMSR	$S\text{–}\chi^2$ (%)
2011	0.892	89544.4(780)***	3.868	0.0	4409.09(697)***	0.039	0.882	0.875	0.049	5.0
2013	0.895	82197.4(780)***	3.760	0.0	3983.61(697)***	0.037	0.901	0.895	0.046	0.0
2015	0.924	87652.4(780)***	4.013	0.0	4135.55(697)***	0.038	0.921	0.917	0.041	2.5

Note. n = 3,456 for each wave; number of frailty index items = 40. KMO = Kaiser–Meyer–Olkin measure of sampling adequacy; Bartlett's χ^2 = Bartlett's test of sphericity; df = degrees of freedom; λ_1/λ_2 = ratio of the first to second eigenvalue, with values >3 indicating a unidimensional approximation; LD = proportion of item pairs showing local dependence ($Q_3 > 0.36$); M_2 = limited-information goodness-of-fit statistic; RMSEA = root mean square error of approximation; CFI = comparative fit index; TLI = Tucker–Lewis index; SRMSR = standardized root mean square residual; $S\text{–}\chi^2$ = proportion of items showing significant misfit based on the Orlando–Thissen $S\text{–}\chi^2$ statistic with Bonferroni correction (α = 0.05/40; $P < 0.00125$). * $P < .05$. ** $P < .01$. *** $P < .001$.

Table S5. Graded Response Model Item Parameters and Fit Statistics Across Waves.

Item	2011								2013								2015							
	a	b_1	b_2	b_3	b_4	b_5	*S-χ²*	*P*	*a*	b_1	b_2	b_3	b_4	b_5	*S-χ²*	*P*	*a*	b_1	b_2	b_3	b_4	b_5	*S-χ²*	*P*
Chronic diseases																								
Hypertension	.398	2.320	—	—	—	—	43.67	.528	.357	2.564	—	—	—	—	38.72	.799	.320	2.859	—	—	—	—	64.07	.164
Dyslipidemia	.332	6.732	—	—	—	—	42.39	.411	.369	6.094	—	—	—	—	57.56	.055	.215	1.305	—	—	—	—	45.27	.544
Diabetes or high blood sugar	.415	6.773	—	—	—	—	33.09	.736	.402	6.990	—	—	—	—	38.73	.528	.238	11.629	—	—	—	—	71.62	.005
Cancer or malignant tumor	.427	11.412	—	—	—	—	17.40	.496	.102	46.357	—	—	—	—	16.44	.562	.197	24.366	—	—	—	—	14.56	.801
Chronic lung disease	.449	4.260	—	—	—	—	55.98	.126	.507	3.806	—	—	—	—	31.85	.914	.355	5.311	—	—	—	—	43.07	.807
Liver disease	.438	7.350	—	—	—	—	57.40	.013	.323	9.831	—	—	—	—	46.52	.075	.262	12.060	—	—	—	—	54.05	.055
Heart disease	.519	3.630	—	—	—	—	47.25	.381	.483	3.874	—	—	—	—	44.71	.442	.417	4.449	—	—	—	—	42.75	.816
Stroke	1.075	3.720	—	—	—	—	32.09	.738	.887	4.333	—	—	—	—	44.47	.218	.721	5.183	—	—	—	—	43.13	.423
Kidney disease	.528	5.200	—	—	—	—	36.43	.632	.356	7.531	—	—	—	—	36.32	.637	.402	6.713	—	—	—	—	32.70	.930
Stomach or digestive disease	.414	2.978	—	—	—	—	56.54	.116	.266	4.544	—	—	—	—	49.78	.325	.189	6.335	—	—	—	—	49.24	.658
Psychiatric/emotional problems	.736	6.013	—	—	—	—	30.93	.320	.523	8.225	—	—	—	—	30.15	.356	.412	1.340	—	—	—	—	23.73	.742
Memory-related disease	.739	5.647	—	—	—	—	35.07	.281	.514	7.866	—	—	—	—	35.71	.218	.549	7.397	—	—	—	—	28.71	.681
Arthritis or rheumatism	.707	.721	—	—	—	—	62.16	.018	.535	.916	—	—	—	—	43.94	.474	.429	1.111	—	—	—	—	64.60	.113
Asthma	.470	6.309	—	—	—	—	45.48	.220	.476	6.238	—	—	—	—	47.61	.162	.312	9.342	—	—	—	—	39.44	.667
Mobility limitations																								
Jogging 1 km	1.344	−.500	−.065	.066	—	—	86.43	.173	1.580	−.503	−.167	−.116	—	—	57.19	.714	1.660	−.632	−.339	−.297	—	—	86.26	.057
Walking 1 km	1.455	1.150	1.982	2.146	—	—	**151.39**	**<.001†**	1.554	.979	1.702	1.789	—	—	85.76	.690	1.607	.650	1.296	1.369	—	—	132.55	.012
Getting up from a chair	1.984	.642	2.477	3.266	—	—	**127.28**	**<.001†**	1.756	.634	2.697	3.304	—	—	133.15	.001	1.802	.456	2.375	3.020	—	—	122.47	.277
Climbing several flights of stairs	1.876	.109	1.242	1.497	—	—	104.50	.176	1.916	.044	1.109	1.272	—	—	95.67	.246	2.159	−.073	.914	1.059	—	—	87.10	.625
Stooping/kneeling/crouching	1.983	.509	1.647	1.895	—	—	98.37	.414	1.825	.424	1.504	1.693	—	—	106.98	.189	1.996	.225	1.180	1.312	—	—	104.91	.323
Reaching arms above shoulder level	1.648	1.753	2.330	2.525	—	—	124.45	.037	1.712	1.638	2.041	2.111	—	—	112.21	.005	1.617	1.469	1.924	1.985	—	—	76.52	.782
Lifting/carrying >10 jin (≈5 kg)	1.925	1.486	1.922	2.052	—	—	92.99	.452	1.941	1.287	1.624	1.706	—	—	94.44	.354	2.175	1.001	1.333	1.430	—	—	77.95	.950

Picking up a small coin from a table	1.702	2.494	3.102	3.320	—	—	54.32	.825	1.542	2.567	3.019	3.098	—	—	60.59	.806	1.664	2.142	2.603	2.683	—	—	78.38	.759
Activities of daily living (ADL)																								
Dressing	2.977	1.887	2.673	3.324	—	—	42.19	.377	2.857	1.784	2.604	3.458	—	—	69.16	.046	2.946	1.572	2.341	2.921	—	—	88.98	.085
Bathing or showering	3.198	1.689	2.251	2.806	—	—	75.49	.086	3.483	1.598	2.000	2.544	—	—	70.52	.394	3.270	1.386	1.820	2.350	—	—	115.79	.030
Eating (e.g., cutting food)	2.737	2.221	2.901	3.452	—	—	29.79	.674	2.720	2.307	3.159	3.563	—	—	42.39	.153	2.732	2.101	2.817	3.318	—	—	50.21	.386
Getting in or out of bed	3.208	1.773	2.708	3.473	—	—	36.00	.330	2.956	1.770	2.812	3.318	—	—	57.33	.007	2.460	1.538	2.757	3.294	—	—	80.18	.028
Using the toilet	2.402	1.308	2.316	2.859	—	—	107.40	.010	2.300	1.252	2.351	2.756	—	—	103.20	.180	2.102	1.081	2.249	2.664	—	—	142.67	.041
Controlling urination/defecation	1.558	2.433	3.459	3.859	—	—	54.39	.680	1.488	2.542	3.442	3.826	—	—	68.55	.425	1.628	2.182	3.072	3.401	—	—	75.85	.751
Instrumental activities of daily living (IADL)																								
Doing household chores	2.947	1.493	2.053	2.240	—	—	96.94	.158	2.898	1.371	1.832	2.032	—	—	91.83	.456	2.950	1.052	1.550	1.766	—	—	**165.91**	**<.001†**
Preparing hot meals	2.671	1.590	2.018	2.176	—	—	87.84	.455	2.717	1.524	1.842	1.995	—	—	84.06	.684	2.963	1.264	1.584	1.736	—	—	95.16	.476
Shopping for groceries	2.203	1.657	2.085	2.328	—	—	119.96	.057	2.430	1.595	1.846	2.001	—	—	101.43	.307	2.584	1.403	1.631	1.823	—	—	109.17	.424
Managing money	1.352	1.706	2.160	2.470	—	—	114.90	.331	1.569	1.751	2.002	2.226	—	—	105.09	.479	1.617	1.567	1.863	2.121	—	—	140.87	.105
Taking medications	1.296	2.465	3.171	4.243	—	—	98.28	.172	1.492	2.507	2.944	3.896	—	—	64.51	.723	1.666	2.207	2.622	3.395	—	—	90.78	.710
Cognition																								
Calculation deficit (serial 7s)	.291	−.617	.635	1.858	3.364	8.825	205.83	.022	.170	−.796	1.612	4.020	6.837	18.593	200.59	.067	.154	−1.555	1.222	3.581	7.536	21.256	215.36	.189
Visuospatial deficit (figure redraw)	.610	.420	—	—	—	—	54.02	.084	.447	.439	—	—	—	—	53.04	.165	.546	−.063	—	—	—	—	59.72	.163
Orientation deficit	.299	1.711	8.854	—	—	—	92.26	.129	.191	5.123	16.538	—	—	—	70.29	.568	.283	2.437	9.561	—	—	—	117.61	.043
Memory deficit (recall ≤ wave median)	.461	1.136	—	—	—	—	35.91	.802	.487	−3.779	—	—	—	—	42.63	.147	.532	.110	—	—	—	—	51.66	.409
Self-rated measures																								
Self-rated health	.892	−.377	—	—	—	—	68.05	.001	.797	−.214	—	—	—	—	48.93	.157	.796	−.388	—	—	—	—	26.10	.989
Self-rated memory	.861	−2.175	—	—	—	—	55.32	.002	.580	−3.297	—	—	—	—	36.44	.312	.602	−3.716	—	—	—	—	57.52	.007
Life satisfaction	.541	3.395	—	—	—	—	53.74	.174	.719	3.025	—	—	—	—	40.68	.694	.913	3.048	—	—	—	—	44.79	.751

Note. *a* = discrimination parameter; *b* = difficulty (threshold) parameter, subscripts indicate category thresholds; $S\text{-}\chi^2$ = Orlando and Thissen's signed chi-square item-fit statistic; *P* = *P*-value for $S\text{-}\chi^2$. "—" indicates the threshold parameter is not applicable for the given item. Items are grouped by domain. Bold $S\text{-}\chi^2$ and *P* values with † indicate statistically significant item misfit using a Bonferroni-corrected threshold ($\alpha = 0.05/40$; $P < 0.00125$).

Table S6. Fit Indices and RMSD-Based DIF Diagnostics for Longitudinal Measurement Invariance of the GRM-Based FI-IRT.

Model	AIC	BIC	CFI	TLI	RMSEA	ΔCFI	ΔRMSEA	Conclusion
Panel A: Sequential model comparison								
Configural invariance	366,021	368,698	.937	.934	.023	—	—	—
Metric invariance (equal discriminations)	366,058	368,169	.937	.935	.023	−.0007	−.0002	Supported
Full scalar invariance (equal discriminations and thresholds)	368,650	369,571	.923	.927	.024	−.0136	+.0014	Not fully supported
Partial scalar invariance	—	—	.933	—	.022	−.0044	−.0003	Supported
Panel B: Items with substantive DIF (RMSD ≥ 0.050)								
**Item**	**RMSD**	**Classification**	**LRT adj. *P***	**LRT**				**Freed**
kneeling	0.050	Medium	< .001	Sig.				Yes
arthritis	0.057	Medium	< .001	Sig.				Yes
orientation_score	0.060	Medium	< .001	Sig.				Yes
memory_deficit	0.284	Large	< .001	Sig.				Yes
Remaining 36 items	0.005–0.049	Negligible–Small	—	—				No (retained as anchors)

Note. Panel A presents sequential model comparisons. Panel B presents items with substantive DIF, defined as RMSD ≥ 0.050. FI-IRT = item response theory-derived frailty index; GRM = graded response model; RMSD = root-mean-square deviation; DIF = differential item functioning; AIC = Akaike information criterion; BIC = Bayesian information criterion; CFI = comparative fit index; TLI = Tucker-Lewis index; RMSEA = root mean square error of approximation. ΔCFI and ΔRMSEA were calculated relative to the configural model. Invariance was considered supported when ΔCFI ≥ -0.010 and ΔRMSEA ≤ 0.015. The metric model constrained item discriminations to equality across waves, and the full scalar model additionally constrained item thresholds. Because full scalar invariance was not supported, item-level DIF was evaluated using RMSD as the primary effect-size criterion. Likelihood-ratio DIF tests are reported as supplementary evidence. After freeing the four RMSD-flagged items, partial scalar invariance was supported.

Table S7. RMSD-Based DIF Diagnostics and Likelihood-Ratio DIF Results for All 40 FI-IRT Items Across Three Waves.

Domain	Item	RMSD	RMSD classification	LRT adjusted *P*	LRT result	Final verdict
Functional limitations	jogging_1km	.026	Small	< .001	Significant	Invariant
	walking_1km	.039	Small	< .001	Significant	Invariant
	chair	.026	Small	.024	Significant	Invariant
	climbing	.035	Small	< .001	Significant	Invariant
	kneeling	.050	Medium	< .001	Significant	**Non-invariant**
	arms	.033	Small	< .001	Significant	Invariant
	carrying	.031	Small	< .001	Significant	Invariant
	picking	.034	Small	.014	Significant	Invariant
ADL	dressing	.024	Small	.879	Not significant	Invariant
	bathing	.020	Small	.289	Not significant	Invariant
	eating	.024	Small	.055	Not significant	Invariant
	out_of_bed	.033	Small	.002	Significant	Invariant
	toilet	.023	Small	.037	Significant	Invariant
	urination	.023	Small	.723	Not significant	Invariant
IADL	household	.021	Small	< .001	Significant	Invariant
	meals	.034	Small	.421	Not significant	Invariant
	shopping	.020	Small	< .001	Significant	Invariant
	money	.041	Small	< .001	Significant	Invariant
	medications	.035	Small	< .001	Significant	Invariant
Chronic diseases	hypertension	.026	Small	.315	Not significant	Invariant
	dyslipidemia	.022	Small	.417	Not significant	Invariant
	diabetes	.013	Negligible	.421	Not significant	Invariant
	cancer	.005	Negligible	.864	Not significant	Invariant
	lung	.024	Small	.175	Not significant	Invariant
	liver	.012	Negligible	.748	Not significant	Invariant
	heart	.024	Small	.287	Not significant	Invariant
	stroke	.034	Small	.095	Not significant	Invariant
	kidney	.014	Negligible	.516	Not significant	Invariant
	stomach	.037	Small	.012	Significant	Invariant

	psychiatric	.011	Negligible	.587	Not significant	Invariant
	memory_related	.015	Negligible	.715	Not significant	Invariant
	arthritis	.057	Medium	< .001	Significant	**Non-invariant**
	asthma	.019	Negligible	.460	Not significant	Invariant
	calculation_score	.036	Small	< .001	Significant	Invariant
	visuospatial	.032	Small	.001	Significant	Invariant
Cognition	orientation_score	.060	Medium	< .001	Significant	**Non-invariant**
	memory_deficit	.284	Large	< .001	Significant	**Non-invariant**
	self_rated_health	.038	Small	< .001	Significant	Invariant
Self-rated measures	self_rated_memory	.041	Small	< .001	Significant	Invariant
	life_satisfaction	.049	Small	< .001	Significant	Invariant

Note. RMSD = maximum root-mean-square deviation across waves and response categories; LRT = likelihood-ratio test with Benjamini-Hochberg correction. RMSD was used as the primary effect-size criterion for item-level non-invariance. Items with RMSD ≥ 0.050 were classified as non-invariant. LRT results are reported as supplementary evidence because significance-based DIF tests can be sensitive in large samples. Items are grouped by frailty domain for readability.

Table S8. Spearman Correlation Coefficients and Distributions of Study Variables.

Variable	Median (IQR)	1	2	3	4	5	6	7	8	9
1. T_1 SRS	0.03 (−0.17, 0.22)	1	—	—	—	—	—	—	—	—
2. T_1 SII	2.00 (1.00, 2.00)	0.13***	1	—	—	—	—	—	—	—
3. T_1 FI-IRT	0.41 (0.26, 0.56)	0.26***	0.15***	1	—	—	—	—	—	—
4. T_2 SRS	0.05 (−0.15, 0.24)	0.48***	0.16***	0.23***	1	—	—	—	—	—
5. T_2 SII	2.00 (1.00, 2.00)	0.17***	0.46***	0.17***	0.19***	1	—	—	—	—
6. T_2 FI-IRT	0.41 (0.27, 0.56)	0.22***	0.11***	0.60***	0.25***	0.13***	1	—	—	—
7. T_3 SRS	0.11 (−0.08, 0.29)	0.51***	0.14***	0.23***	0.59***	0.19***	0.26***	1	—	—
8. T_3 SII	2.00 (1.00, 2.00)	0.14***	0.42***	0.15***	0.19***	0.45***	0.13***	0.17***	1	—
9. T_3 FI-IRT	0.39 (0.25, 0.53)	0.25***	0.11***	0.58***	0.28***	0.14***	0.64***	0.32***	0.17***	1

Note. T_1 = Wave 1 (2011); T_2 = Wave 2 (2013); T_3 = Wave 3 (2015). SRS = Sarcopenia Risk Score; SII = Social Isolation Index; FI-IRT = Item response theory-derived frailty index. IQR = interquartile range. Correlations are Spearman's ρ. *$P < .05$; **$P < .01$; ***$P < .001$.

Table S9. Standardized Path Coefficients for the Four Best-Fitting Panel Models.

Parameter	Model 1a		Model 2d		Model 3d		Model 4d[a]	
	β (*SE*)	*P*	*β* (*SE*)	*P*	*β* (*SE*)	*P*	*β* (*SE*)	*P*
**Model Fit Indices**								
χ^2 (*df*)	575.2 (9)		954.9 (105)		99.4 (39)		482.7 (123)	
RMSEA [90% CI]	0.166 [0.156, 0.176]		0.052 [0.049, 0.055]		0.022 [0.017, 0.028]		0.031 [0.028, 0.033]	
CFI	0.917		0.918		0.994		0.967	
TLI	0.334		0.860		0.988		0.952	
SRMR	0.033		0.024		0.014		0.027	
**Between-Person Relations**								
$RI_{SRS} \leftrightarrow RI_{SII}$	—	—	—	—	0.187*** (0.037)	<.001	0.131** (0.041)	.001
$RI_{SRS} \leftrightarrow RI_{FI\text{-}IRT}$	—	—	—	—	0.364*** (0.033)	<.001	0.245*** (0.055)	<.001
$RI_{SII} \leftrightarrow RI_{FI\text{-}IRT}$	—	—	—	—	0.152*** (0.037)	<.001	−0.076 (0.069)	.272
**Autoregressive Paths**								
SRS $T_1 \rightarrow$ SRS T_2	0.401*** (0.016)	<.001	0.421*** (0.015)	<.001	0.014 (0.022)	.522	0.021 (0.022)	.348
SRS $T_2 \rightarrow$ SRS T_3	0.456*** (0.027)	<.001	0.431*** (0.026)	<.001	0.014 (0.022)	.529	0.021 (0.023)	.360
SII $T_1 \rightarrow$ SII T_2	0.440*** (0.015)	<.001	0.439*** (0.013)	<.001	0.043* (0.021)	.036	0.055* (0.022)	.012
SII $T_2 \rightarrow$ SII T_3	0.443*** (0.017)	<.001	0.439*** (0.014)	<.001	0.043* (0.021)	.040	0.055* (0.022)	.014
FI-IRT $T_1 \rightarrow$ FI-IRT T_2	0.558*** (0.014)	<.001	0.495*** (0.012)	<.001	0.079*** (0.018)	<.001	0.235*** (0.031)	<.001
FI-IRT $T_2 \rightarrow$ FI-IRT T_3	0.586*** (0.014)	<.001	0.493*** (0.013)	<.001	0.082*** (0.019)	<.001	0.241*** (0.031)	<.001
**Cross-Lagged Paths**								
SRS ↔ SII								
T_1 SRS $\rightarrow$ T_2 SII	0.071*** (0.017)	<.001	0.071*** (0.011)	<.001	0.039* (0.016)	.012	0.039* (0.016)	.014
T_2 SRS $\rightarrow$ T_3 SII	0.077*** (0.016)	<.001	0.069*** (0.011)	<.001	0.037* (0.015)	.012	0.037* (0.015)	.013
T_1 SII $\rightarrow$ T_2 SRS	0.073*** (0.015)	<.001	0.065*** (0.010)	<.001	0.030* (0.014)	.031	0.043** (0.014)	.003
T_2 SII $\rightarrow$ T_3 SRS	0.061*** (0.014)	<.001	0.069*** (0.011)	<.001	0.031* (0.014)	.031	0.044** (0.015)	.003
SRS ↔ FI-IRT								
T_1 SRS $\rightarrow$ T_2 FI-IRT	0.060*** (0.015)	<.001	0.071*** (0.010)	<.001	0.027 (0.014)	.052	0.037* (0.017)	.025
T_2 SRS $\rightarrow$ T_3 FI-IRT	0.106*** (0.015)	<.001	0.072*** (0.010)	<.001	0.027 (0.014)	.052	0.037* (0.017)	.025

T_1 FI-IRT → T_2 SRS	0.073*** (0.017)	<.001	0.075*** (0.012)	<.001	0.026 (0.016)	.108	0.070*** (0.019)	<.001
T_2 FI-IRT → T_3 SRS	0.127*** (0.018)	<.001	0.076*** (0.013)	<.001	0.027 (0.017)	.114	0.072*** (0.020)	<.001
SII ↔ FI-IRT								
T_1 SII → T_2 FI-IRT	0.012 (0.014)	.115	0.007 (0.009)	.463	−0.006 (0.013)	.618	0.037* (0.017)	.030
T_2 SII → T_3 FI-IRT	0.017 (0.014)	.116	0.007 (0.009)	.464	−0.007 (0.014)	.618	0.038* (0.018)	.029
T_1 FI-IRT → T_2 SII	0.050** (0.016)	<.001	0.022 (0.012)	.057	0.023 (0.016)	.161	0.072*** (0.021)	<.001
T_2 FI-IRT → T_3 SII	0.035* (0.017)	<.001	0.022 (0.011)	.058	0.023 (0.016)	.161	0.071*** (0.021)	<.001
**Residual Correlations**								
T_1: SRS ↔ SII	0.090*** (0.017)	<.001	0.071*** (0.017)	<.001	0.017 (0.026)	.511	0.025 (0.026)	.339
T_1: SRS ↔ FI-IRT	0.244*** (0.017)	<.001	0.193*** (0.018)	<.001	0.153*** (0.025)	<.001	0.166*** (0.026)	<.001
T_1: SII ↔ FI-IRT	0.109*** (0.018)	<.001	0.053** (0.018)	.003	0.054* (0.026)	.035	0.092** (0.030)	.002
T_2: SRS ↔ SII	0.047** (0.017)	.087	0.017 (0.013)	.204	−0.005 (0.019)	.808	−0.001 (0.019)	.957
T_2: SRS ↔ FI-IRT	0.116*** (0.019)	<.001	0.108*** (0.014)	<.001	0.072*** (0.022)	<.001	0.081*** (0.022)	<.001
T_2: SII ↔ FI-IRT	0.014 (0.018)	<.001	0.026* (0.013)	.037	0.038* (0.018)	.035	0.061** (0.019)	.001
T_3: SRS ↔ SII	-0.002 (0.022)	.101	0.017 (0.013)	.219	−0.004 (0.018)	.806	−0.001 (0.018)	.957
T_3: SRS ↔ FI-IRT	0.146*** (0.025)	<.001	0.116*** (0.020)	<.001	0.076** (0.027)	.004	0.090** (0.029)	.002
T_3: SII ↔ FI-IRT	0.083*** (0.018)	<.001	0.027* (0.013)	.038	0.037* (0.018)	.036	0.063** (0.020)	.001

Note. *β* = standardized path coefficient; *SE* = standard error; SRS = Sarcopenia Risk Score; SII = Social Isolation Index; FI-IRT = Item response theory-derived frailty index; TIC = time-invariant covariates; TVC = time-varying covariates; RI = random intercept; CLPM = cross-lagged panel model; RI-CLPM = random-intercept cross-lagged panel model. Model 1a: unconstrained CLPM with TIC; Model 2d: stationarity-constrained CLPM with TIC and TVC; Model 3d: stationarity-constrained RI-CLPM with TIC; Model 4d: stationarity-constrained RI-CLPM with TIC and TVC (primary analysis). TIC included age, sex, educational attainment, and residence. TVC included depressive symptoms, smoking status, drinking status, and sleep quality. Random-intercept parameters are not applicable to the CLPM models. Coefficients are from the standardized solution (std.all).

$^*P < .05$. $^{**}P < .01$. $^{***}P < .001$.

Table S10. Model Fit and Comparisons for Multigroup Random-Intercept Cross-Lagged Panel Models.

Models	Model Fit				Model Comparison		
	$\chi^2(df)$	CFI	RMSEA [90% CI]	SRMR	ΔCFI	ΔRMSEA	ΔSRMR
**Age**							
Model 1	621.287(234)***	0.963	0.032 [0.028, 0.035]	0.030	—	—	—
Model 2	635.353(255)***	0.964	0.030 [0.027, 0.033]	0.030	0.001	0.002	0.000
**Sex**							
Model 1	584.804(234)***	0.967	0.031 [0.027, 0.034]	0.030	—	—	—
Model 2	616.056(255)***	0.966	0.030 [0.027, 0.033]	0.031	0.001	0.001	0.001
**Education**							
Model 1	585.396(234)***	0.966	0.031 [0.027, 0.034]	0.029	—	—	—
Model 2	607.989(255)***	0.966	0.030 [0.026, 0.033]	0.030	0.001	0.001	0.000
**Residence**							
Model 1	604.975(234)***	0.966	0.031 [0.028, 0.035]	0.029	—	—	—
Model 2	629.426(255)***	0.965	0.030 [0.027, 0.034]	0.029	0.001	0.001	0.000

Note. RI-CLPM = random-intercept cross-lagged panel model; χ^2 = Satorra-Bentler scaled chi-square; *df* = degrees of freedom; CFI = comparative fit index; RMSEA = root mean square error of approximation; CI = confidence interval; SRMR = standardized root mean squared residual; Δ = absolute change in fit index. Model 1 denotes the unconstrained multigroup RI-CLPM, whereas Model 2 denotes the constrained multigroup RI-CLPM with structural paths constrained to be equal across groups. *** $P < .001$.

Table S11. Multigroup Random-Intercept Cross-Lagged Panel Model Results by Age, CHARLS, 2011–2015.

Parameter	Younger (n = 2,751)		Older (n = 408)	
	B (SE)	β (SE)	B (SE)	β (SE)
Model Fit Indices				
$\chi^2(df)$	635.4(255)***			
RMSEA [90% CI]	0.030 [0.027, 0.033]			
CFI	0.964			
TLI	0.952			
SRMR	0.030			
Between-Person Relations				
$RI_{SRS} \leftrightarrow RI_{SII}$	0.014(0.004)***	0.156(0.043)***	0.012(0.007)	0.136(0.080)
$RI_{SRS} \leftrightarrow RI_{FI\text{-}IRT}$	0.003(0.001)***	0.234(0.056)***	0.004(0.001)**	0.396(0.112)**
$RI_{SII} \leftrightarrow RI_{FI\text{-}IRT}$	−0.003(0.002)	−0.082(0.069)	−0.002(0.003)	−0.063(0.124)
Autoregressive Paths				
SRS T_1 → SRS T_2	0.024(0.020)	0.024(0.020)	0.024(0.020)	0.026(0.021)
SRS T_2 → SRS T_3	0.024(0.020)	0.025(0.020)	0.024(0.020)	0.021(0.018)
SII T_1 → SII T_2	0.054(0.023)*	0.053(0.022)*	0.054(0.023)*	0.054(0.023)*
SII T_2 → SII T_3	0.054(0.023)*	0.053(0.022)*	0.054(0.023)*	0.050(0.022)*
FI-IRT T_1 → FI-IRT T_2	0.240(0.031)***	0.233(0.031)***	0.240(0.031)***	0.254(0.035)***
FI-IRT T_2 → FI-IRT T_3	0.240(0.031)***	0.241(0.031)***	0.240(0.031)***	0.244(0.033)***
Cross-Lagged Paths				
**SRS ↔ SII**				
T_1 SRS → T_2 SII	0.084(0.042)*	0.032(0.016)*	0.084(0.042)*	0.031(0.015)*
T_2 SRS → T_3 SII	0.084(0.042)*	0.031(0.015)*	0.084(0.042)*	0.027(0.013)*
T_1 SII → T_2 SRS	0.016(0.006)**	0.040(0.015)**	0.016(0.006)**	0.045(0.016)**
T_2 SII → T_3 SRS	0.016(0.006)**	0.042(0.016)**	0.016(0.006)**	0.038(0.015)**
**SRS ↔ FI-IRT**				
T_1 SRS → T_2 FI-IRT	0.019(0.008)*	0.038(0.017)*	0.019(0.008)*	0.039(0.017)*
T_2 SRS → T_3 FI-IRT	0.019(0.008)*	0.037(0.017)*	0.019(0.008)*	0.037(0.017)*
T_1 FI-IRT → T_2 SRS	0.138(0.042)***	0.068(0.021)***	0.138(0.042)***	0.076(0.023)***
T_2 FI-IRT → T_3 SRS	0.138(0.042)***	0.072(0.023)***	0.138(0.042)***	0.061(0.022)***
**SII ↔ FI-IRT**				
T_1 SII → T_2 FI-IRT	0.007(0.003)*	0.038(0.017)*	0.007(0.003)*	0.041(0.019)*
T_2 SII → T_3 FI-IRT	0.007(0.003)*	0.039(0.018)*	0.007(0.003)*	0.042(0.019)*
T_1 FI-IRT → T_2 SII	0.387(0.114)***	0.072(0.021)***	0.387(0.114)***	0.074(0.022)***
T_2 FI-IRT → T_3 SII	0.387(0.114)***	0.071(0.021)***	0.387(0.114)***	0.064(0.019)***
Residual Correlations				
T_1: SRS ↔ SII	0.002(0.004)	0.015(0.028)	0.005(0.009)	0.032(0.063)

T_1: SRS ↔ FI-IRT	0.004(0.001)***	0.170(0.028)***	0.004(0.002)	0.123(0.061)
T_1: SII ↔ FI-IRT	0.004(0.002)*	0.071(0.031)*	0.017(0.005)***	0.216(0.059)***
T_2: SRS ↔ SII	0.001(0.002)	0.004(0.019)	−0.003(0.006)	−0.022(0.050)
T_2: SRS ↔ FI-IRT	0.002(0.000)***	0.097(0.019)***	−0.000(0.002)	−0.005(0.069)
T_2: SII ↔ FI-IRT	0.003(0.001)*	0.049(0.020)*	0.008(0.003)**	0.133(0.046)**
T_3: SRS ↔ SII	0.001(0.002)	0.005(0.019)	−0.003(0.006)	−0.015(0.032)
T_3: SRS ↔ FI-IRT	0.002(0.000)***	0.115(0.025)***	−0.000(0.002)	−0.004(0.058)
T_3: SII ↔ FI-IRT	0.003(0.001)*	0.052(0.021)*	0.008(0.003)**	0.127(0.045)**

Note. *B* = unstandardized coefficient; *SE* = standard error; β = standardized coefficient; SRS = Sarcopenia Risk Score; SII = Social Isolation Index; FI-IRT = item response theory-derived frailty index; RI = random intercept. T_1, T_2, and T_3 denote Waves 1, 2, and 3 (2011, 2013, and 2015), respectively. Age groups were defined as 60–74 years and ≥75 years. Unstandardized structural path coefficients were constrained to equality across age groups, whereas standardized coefficients are presented separately for each group. This constraint was supported by the multigroup invariance results (ΔCFI ≤ 0.010, ΔRMSEA ≤ 0.015, and ΔSRMR ≤ 0.010). *$P < .05$. **$P < .01$. ***$P < .001$.

Table S12. Multigroup Random-Intercept Cross-Lagged Panel Model Results by Sex, CHARLS, 2011–2015.

Parameter	Female (n = 1,641)		Male (n = 1,516)	
	B (SE)	β (SE)	B (SE)	β (SE)
Model Fit Indices				
χ^2(df)	616.1(255)***			
RMSEA [90% CI]	0.030 [0.027, 0.033]			
CFI	0.966			
TLI	0.954			
SRMR	0.031			
Between-Person Relations				
$RI_{SRS} \leftrightarrow RI_{SII}$	0.009(0.005)*	0.097(0.048)*	0.014(0.004)***	0.184(0.051)***
$RI_{SRS} \leftrightarrow RI_{FI\text{-}IRT}$	0.004(0.001)***	0.248(0.058)***	0.002(0.001)**	0.229(0.072)**
$RI_{SII} \leftrightarrow RI_{FI\text{-}IRT}$	−0.003(0.002)	−0.099(0.078)	−0.002(0.002)	−0.081(0.082)
Autoregressive Paths				
SRS T_1 → SRS T_2	0.019(0.023)	0.019(0.023)	0.019(0.023)	0.018(0.022)
SRS T_2 → SRS T_3	0.019(0.023)	0.019(0.024)	0.019(0.023)	0.018(0.022)
SII T_1 → SII T_2	0.057(0.023)*	0.056(0.022)*	0.057(0.023)*	0.055(0.022)*
SII T_2 → SII T_3	0.057(0.023)*	0.055(0.022)*	0.057(0.023)*	0.055(0.022)*
FI-IRT T_1 → FI-IRT T_2	0.248(0.033)***	0.242(0.033)***	0.248(0.033)***	0.244(0.034)***
FI-IRT T_2 → FI-IRT T_3	0.248(0.033)***	0.251(0.034)***	0.248(0.033)***	0.250(0.033)***
Cross-Lagged Paths				
**SRS ↔ SII**				
T_1 SRS → T_2 SII	0.103(0.041)*	0.044(0.017)*	0.103(0.041)*	0.035(0.014)*
T_2 SRS → T_3 SII	0.103(0.041)*	0.041(0.016)*	0.103(0.041)*	0.035(0.014)*
T_1 SII → T_2 SRS	0.018(0.006)**	0.043(0.013)**	0.018(0.006)**	0.050(0.016)**
T_2 SII → T_3 SRS	0.018(0.006)**	0.046(0.014)**	0.018(0.006)**	0.050(0.016)**
**SRS ↔ FI-IRT**				
T_1 SRS → T_2 FI-IRT	0.019(0.008)*	0.043(0.019)*	0.019(0.008)*	0.034(0.015)*
T_2 SRS → T_3 FI-IRT	0.019(0.008)*	0.042(0.018)*	0.019(0.008)*	0.036(0.016)*
T_1 FI-IRT → T_2 SRS	0.155(0.040)***	0.068(0.017)***	0.155(0.040)***	0.080(0.021)***
T_2 FI-IRT → T_3 SRS	0.155(0.040)***	0.072(0.019)***	0.155(0.040)***	0.077(0.021)***
**SII ↔ FI-IRT**				
T_1 SII → T_2 FI-IRT	0.008(0.003)*	0.040(0.018)*	0.008(0.003)*	0.039(0.018)*
T_2 SII → T_3 FI-IRT	0.008(0.003)*	0.041(0.018)*	0.008(0.003)*	0.041(0.019)*
T_1 FI-IRT → T_2 SII	0.394(0.118)***	0.072(0.022)***	0.394(0.118)***	0.072(0.022)***
T_2 FI-IRT → T_3 SII	0.394(0.118)***	0.071(0.021)***	0.394(0.118)***	0.070(0.021)***
Residual Correlations				
T_1: SRS ↔ SII	0.006(0.005)	0.042(0.033)	0.001(0.005)	0.006(0.037)

T_1: SRS ↔ FI-IRT	0.005(0.001)***	0.184(0.032)***	0.004(0.001)***	0.151(0.036)***
T_1: SII ↔ FI-IRT	0.007(0.002)**	0.104(0.036)**	0.006(0.003)*	0.089(0.039)*
T_2: SRS ↔ SII	0.004(0.003)	0.032(0.022)	−0.004(0.003)	−0.033(0.027)
T_2: SRS ↔ FI-IRT	0.002(0.001)***	0.086(0.023)***	0.002(0.001)**	0.083(0.031)**
T_2: SII ↔ FI-IRT	0.003(0.002)*	0.052(0.025)*	0.004(0.001)**	0.070(0.024)**
T_3: SRS ↔ SII	0.004(0.003)	0.035(0.024)	−0.004(0.003)	−0.029(0.022)
T_3: SRS ↔ FI-IRT	0.002(0.001)***	0.108(0.029)***	0.002(0.001)**	0.085(0.038)**
T_3: SII ↔ FI-IRT	0.003(0.002)*	0.055(0.026)*	0.004(0.001)**	0.072(0.025)**

Note. *B* = unstandardized coefficient; *SE* = standard error; β = standardized coefficient; SRS = Sarcopenia Risk Score; SII = Social Isolation Index; FI-IRT = item response theory-derived frailty index; RI = random intercept. T_1, T_2, and T_3 denote Waves 1, 2, and 3 (2011, 2013, and 2015), respectively. Sex groups were defined as female and male. Unstandardized structural path coefficients were constrained to equality across sex groups, whereas standardized coefficients are presented separately for each group. This constraint was supported by the multigroup invariance results (ΔCFI ≤ 0.010, ΔRMSEA ≤ 0.015, and ΔSRMR ≤ 0.010). *$P < .05$. **$P < .01$. ***$P < .001$.

Table S13. Multigroup Random-Intercept Cross-Lagged Panel Model Results by Educational Attainment, CHARLS, 2011–2015.

Parameter	Low/Mid Education (*n* = 2,612)		High Education (*n* = 545)	
	B (*SE*)	*β* (*SE*)	*B* (*SE*)	*β* (*SE*)
Model Fit Indices				
$\chi^2(df)$		608.0(255)***		
RMSEA [90% CI]		0.030 [0.026, 0.033]		
CFI		0.966		
TLI		0.954		
SRMR		0.030		
Between-Person Relations				
$RI_{SRS} \leftrightarrow RI_{SII}$	0.010(0.004)**	0.120(0.043)**	0.016(0.007)*	0.174(0.070)*
$RI_{SRS} \leftrightarrow RI_{FI\text{-}IRT}$	0.003(0.001)***	0.239(0.061)***	0.002(0.001)	0.169(0.080)
$RI_{SII} \leftrightarrow RI_{FI\text{-}IRT}$	−0.003(0.002)	−0.085(0.078)	−0.006(0.003)*	−0.183(0.095)*
Autoregressive Paths				
SRS T_1 → SRS T_2	0.024(0.022)	0.024(0.022)	0.024(0.022)	0.026(0.023)
SRS T_2 → SRS T_3	0.024(0.022)	0.024(0.022)	0.024(0.022)	0.027(0.025)
SII T_1 → SII T_2	0.055(0.024)*	0.054(0.023)*	0.055(0.024)*	0.056(0.024)*
SII T_2 → SII T_3	0.055(0.024)*	0.053(0.023)*	0.055(0.024)*	0.054(0.023)*
FI-IRT T_1 → FI-IRT T_2	0.253(0.033)***	0.246(0.033)***	0.253(0.033)***	0.249(0.033)***
FI-IRT T_2 → FI-IRT T_3	0.253(0.033)***	0.253(0.033)***	0.253(0.033)***	0.256(0.034)***
Cross-Lagged Paths				
**SRS ↔ SII**				
T_1 SRS → T_2 SII	0.102(0.041)*	0.039(0.016)*	0.102(0.041)*	0.041(0.017)*
T_2 SRS → T_3 SII	0.102(0.041)*	0.038(0.015)*	0.102(0.041)*	0.038(0.015)*
T_1 SII → T_2 SRS	0.018(0.006)**	0.046(0.014)**	0.018(0.006)**	0.048(0.015)**
T_2 SII → T_3 SRS	0.018(0.006)**	0.046(0.015)**	0.018(0.006)**	0.052(0.016)**
**SRS ↔ FI-IRT**				
T_1 SRS → T_2 FI-IRT	0.021(0.008)**	0.043(0.017)**	0.021(0.008)**	0.047(0.018)**
T_2 SRS → T_3 FI-IRT	0.021(0.008)**	0.043(0.017)**	0.021(0.008)**	0.045(0.017)**
T_1 FI-IRT → T_2 SRS	0.156(0.041)***	0.075(0.020)***	0.156(0.041)***	0.074(0.020)***
T_2 FI-IRT → T_3 SRS	0.156(0.041)***	0.076(0.021)***	0.156(0.041)***	0.082(0.022)***
**SII ↔ FI-IRT**				
T_1 SII → T_2 FI-IRT	0.008(0.003)*	0.043(0.018)*	0.008(0.003)*	0.047(0.020)*
T_2 SII → T_3 FI-IRT	0.008(0.003)*	0.045(0.018)*	0.008(0.003)*	0.047(0.019)*
T_1 FI-IRT → T_2 SII	0.412(0.119)***	0.077(0.023)***	0.412(0.119)***	0.074(0.022)***
T_2 FI-IRT → T_3 SII	0.412(0.119)***	0.075(0.022)***	0.412(0.119)***	0.073(0.021)***
Residual Correlations				
T_1: SRS ↔ SII	0.002(0.004)	0.013(0.028)	0.015(0.009)	0.098(0.056)

T_1: SRS ↔ FI-IRT	0.004(0.001)***	0.158(0.028)***	0.006(0.001)***	0.230(0.050)***
T_1: SII ↔ FI-IRT	0.005(0.002)*	0.084(0.033)*	0.011(0.004)**	0.164(0.056)**
T_2: SRS ↔ SII	0.001(0.003)	0.005(0.022)	−0.004(0.004)	−0.034(0.036)
T_2: SRS ↔ FI-IRT	0.002(0.001)***	0.081(0.024)***	0.002(0.001)**	0.118(0.036)**
T_2: SII ↔ FI-IRT	0.003(0.001)**	0.056(0.021)**	0.007(0.002)**	0.125(0.039)**
T_3: SRS ↔ SII	0.001(0.003)	0.005(0.020)	−0.004(0.004)	−0.041(0.043)
T_3: SRS ↔ FI-IRT	0.002(0.001)***	0.088(0.030)***	0.002(0.001)**	0.165(0.050)**
T_3: SII ↔ FI-IRT	0.003(0.001)**	0.057(0.021)**	0.007(0.002)**	0.132(0.041)**

Note. *B* = unstandardized coefficient; *SE* = standard error; β = standardized coefficient; SRS = Sarcopenia Risk Score; SII = Social Isolation Index; FI-IRT = item response theory-derived frailty index; RI = random intercept. T_1, T_2, and T_3 denote Waves 1, 2, and 3 (2011, 2013, and 2015), respectively. Education groups were defined as junior high school or below and high school or above. Unstandardized structural path coefficients were constrained to equality across education groups, whereas standardized coefficients are presented separately for each group. This constraint was supported by the multigroup invariance results (ΔCFI ≤ 0.010, ΔRMSEA ≤ 0.015, and ΔSRMR ≤ 0.010). *$P < .05$. **$P < .01$. ***$P < .001$.

Table S14. Multigroup Random-Intercept Cross-Lagged Panel Model Results by Residence, CHARLS, 2011–2015.

Parameter	Rural (n = 2,681)		Urban (n = 478)	
	B (SE)	β (SE)	B (SE)	β (SE)
Model Fit Indices				
$\chi^2(df)$	629.4(255)***			
RMSEA [90% CI]	0.030 [0.027, 0.034]			
CFI	0.965			
TLI	0.954			
SRMR	0.029			
Between-Person Relations				
$RI_{SRS} \leftrightarrow RI_{SII}$	0.010(0.004)**	0.118(0.043)**	0.016(0.008)*	0.160(0.071)*
$RI_{SRS} \leftrightarrow RI_{FI\text{-}IRT}$	0.003(0.001)***	0.251(0.057)***	0.002(0.001)	0.144(0.084)
$RI_{SII} \leftrightarrow RI_{FI\text{-}IRT}$	−0.003(0.002)	−0.109(0.075)	−0.000(0.004)	−0.011(0.092)
Autoregressive Paths				
SRS T_1 → SRS T_2	0.013(0.023)	0.013(0.023)	0.013(0.023)	0.014(0.023)
SRS T_2 → SRS T_3	0.013(0.023)	0.013(0.022)	0.013(0.023)	0.015(0.025)
SII T_1 → SII T_2	0.062(0.023)**	0.060(0.022)**	0.062(0.023)**	0.062(0.023)**
SII T_2 → SII T_3	0.062(0.023)**	0.059(0.023)**	0.062(0.023)**	0.060(0.023)**
FI-IRT T_1 → FI-IRT T_2	0.243(0.032)***	0.237(0.032)***	0.243(0.032)***	0.240(0.033)***
FI-IRT T_2 → FI-IRT T_3	0.243(0.032)***	0.244(0.032)***	0.243(0.032)***	0.241(0.031)***
Cross-Lagged Paths				
**SRS ↔ SII**				
T_1 SRS → T_2 SII	0.103(0.041)*	0.041(0.016)*	0.103(0.041)*	0.039(0.016)*
T_2 SRS → T_3 SII	0.103(0.041)*	0.039(0.015)*	0.103(0.041)*	0.036(0.014)*
T_1 SII → T_2 SRS	0.017(0.006)**	0.042(0.014)**	0.017(0.006)**	0.047(0.016)**
T_2 SII → T_3 SRS	0.017(0.006)**	0.043(0.014)**	0.017(0.006)**	0.053(0.018)**
**SRS ↔ FI-IRT**				
T_1 SRS → T_2 FI-IRT	0.019(0.008)*	0.039(0.017)*	0.019(0.008)*	0.041(0.017)*
T_2 SRS → T_3 FI-IRT	0.019(0.008)*	0.039(0.017)*	0.019(0.008)*	0.039(0.016)*
T_1 FI-IRT → T_2 SRS	0.154(0.040)***	0.075(0.020)***	0.154(0.040)***	0.075(0.020)***
T_2 FI-IRT → T_3 SRS	0.154(0.040)***	0.075(0.020)***	0.154(0.040)***	0.086(0.022)***
**SII ↔ FI-IRT**				
T_1 SII → T_2 FI-IRT	0.008(0.003)*	0.039(0.017)*	0.008(0.003)*	0.044(0.019)*
T_2 SII → T_3 FI-IRT	0.008(0.003)*	0.041(0.018)*	0.008(0.003)*	0.043(0.019)*
T_1 FI-IRT → T_2 SII	0.396(0.116)***	0.075(0.022)***	0.396(0.116)***	0.070(0.021)***
T_2 FI-IRT → T_3 SII	0.396(0.116)***	0.074(0.021)***	0.396(0.116)***	0.069(0.020)***
Residual Correlations				
T_1: SRS ↔ SII	−0.001(0.004)	−0.008(0.028)	0.028(0.010)**	0.173(0.058)**

T_1: SRS ↔ FI-IRT	0.004(0.001)***	0.164(0.028)***	0.005(0.002)***	0.198(0.053)***
T_1: SII ↔ FI-IRT	0.006(0.002)**	0.096(0.032)**	0.006(0.004)	0.078(0.058)
T_2: SRS ↔ SII	−0.001(0.003)	−0.009(0.022)	0.004(0.005)	0.030(0.036)
T_2: SRS ↔ FI-IRT	0.002(0.001)***	0.080(0.023)***	0.003(0.001)**	0.118(0.041)**
T_2: SII ↔ FI-IRT	0.004(0.001)**	0.062(0.020)**	0.005(0.002)*	0.078(0.039)*
T_3: SRS ↔ SII	−0.001(0.003)	−0.008(0.019)	0.004(0.005)	0.039(0.047)
T_3: SRS ↔ FI-IRT	0.002(0.001)***	0.085(0.029)***	0.003(0.001)**	0.180(0.058)**
T_3: SII ↔ FI-IRT	0.004(0.001)**	0.064(0.021)**	0.005(0.002)*	0.082(0.042)*

Note. *B* = unstandardized coefficient; *SE* = standard error; β = standardized coefficient; SRS = Sarcopenia Risk Score; SII = Social Isolation Index; FI-IRT = item response theory-derived frailty index; RI = random intercept. T_1, T_2, and T_3 denote Waves 1, 2, and 3 (2011, 2013, and 2015), respectively. Residence groups were defined as rural and urban. Unstandardized structural path coefficients were constrained to equality across residence groups, whereas standardized coefficients are presented separately for each group. This constraint was supported by the multigroup invariance results (ΔCFI ≤ 0.010, ΔRMSEA ≤ 0.015, and ΔSRMR ≤ 0.010). **P* < .05. ***P* < .01. ****P* < .001.

Table S15. Sensitivity Analyses of Cross-Lagged Path Estimates Across Alternative Model Specifications.

Paths	Model 4d[a] (GRM)		Model 5[a] (Deficit Accumulation FI)		Model 6[a] (LL)		Model 7[a,d] (MI Imputation)		Model 8[a,c] (Excl. Baseline Frailty)	
	β (SE)	*P*	β (SE)	*P*	β (SE)	*P*	β (SE)	*P*	β (SE)	*P*
Model Fit Indices										
χ^2 (*df*)	452.2 (123)		544.0 (123)		352.1 (123)		474.6 (123)		348.5 (123)	
RMSEA [90% CI]	0.029 [0.026, 0.032]		0.033 [0.030, 0.036]		0.025 [0.022, 0.028]		0.031 [0.028, 0.034]		0.025 [0.022, 0.028]	
CFI	0.970		0.967		0.976		0.970		0.977	
TLI	0.957		0.951		0.964		0.956		0.966	
SRMR	0.026		0.031		0.023		0.027		0.022	
Cross-Lagged Paths										
T_1 SRS → T_2 SII	0.039 (0.040)	**0.014**	0.037 (0.041)	**0.020**	0.043 (0.040)	**0.006**	0.055 (0.026)	**0.002**	0.038 (0.042)	**0.019**
T_2 SRS → T_3 SII	0.037 (0.040)	**0.014**	0.035 (0.041)	**0.020**	0.041 (0.040)	**0.006**	0.054 (0.026)	**0.002**	0.036 (0.042)	**0.019**
T_1 SII → T_2 SRS	0.041 (0.006)	**0.004**	0.040 (0.006)	**0.005**	0.040 (0.006)	**0.004**	0.051 (0.010)	**<0.001**	0.036 (0.006)	**0.011**
T_2 SII → T_3 SRS	0.042 (0.006)	**0.004**	0.041 (0.006)	**0.005**	0.042 (0.006)	**0.004**	0.053 (0.010)	**<0.001**	0.037 (0.006)	**0.011**
T_1 SII → T_2 FI-IRT	0.034 (0.004)	**0.040**	0.041 (0.002)	**0.014**	0.020 (0.002)	0.238	0.036 (0.004)	**0.029**	0.032 (0.004)	**0.042**
T_2 SII → T_3 FI-IRT	0.035 (0.004)	**0.040**	0.039 (0.002)	**0.014**	0.019 (0.002)	0.238	0.037 (0.004)	**0.029**	0.033 (0.004)	**0.042**
T_1 FI-IRT → T_2 SII	0.069 (0.092)	**<0.001**	0.084 (0.244)	**<0.001**	0.048 (0.165)	**0.011**	0.062 (0.094)	**0.003**	0.058 (0.091)	**0.001**
T_2 FI-IRT → T_3 SII	0.068 (0.092)	**<0.001**	0.083 (0.244)	**<0.001**	0.045 (0.165)	**0.011**	0.061 (0.094)	**0.003**	0.063 (0.091)	**0.001**
T_1 SRS → T_2 FI-IRT	0.034 (0.010)	**0.038**	0.046 (0.004)	**0.003**	0.047 (0.005)	**0.007**	0.051 (0.006)	**0.003**	0.053 (0.009)	**<0.001**
T_2 SRS → T_3 FI-IRT	0.033 (0.010)	**0.038**	0.042 (0.004)	**0.003**	0.043 (0.005)	**0.007**	0.052 (0.006)	**0.003**	0.052 (0.009)	**<0.001**
T_1 FI-IRT → T_2 SRS	0.068 (0.032)	**<0.001**	0.069 (0.089)	**0.003**	0.072 (0.065)	**<0.001**	0.079 (0.054)	**<0.001**	0.066 (0.033)	**<0.001**
T_2 FI-IRT → T_3 SRS	0.070 (0.032)	**<0.001**	0.071 (0.089)	**0.003**	0.070 (0.065)	**<0.001**	0.082 (0.054)	**<0.001**	0.073 (0.033)	**<0.001**
Autoregressive Paths										
T_1 SRS → T_2 SRS	0.018 (0.022)	0.406	0.021 (0.022)	0.352	0.023 (0.022)	0.302	0.077 (0.021)	**<0.001**	0.018 (0.023)	0.419
T_2 SRS → T_3 SRS	0.018 (0.022)	0.406	0.021 (0.022)	0.352	0.023 (0.022)	0.302	0.079 (0.021)	**<0.001**	0.018 (0.023)	0.419
T_1 SII → T_2 SII	0.057 (0.023)	**0.010**	0.057 (0.023)	**0.012**	0.056 (0.023)	**0.011**	0.060 (0.024)	**0.010**	0.067 (0.024)	**0.003**
T_2 SII → T_3 SII	0.057 (0.023)	**0.010**	0.056 (0.023)	**0.012**	0.056 (0.023)	**0.011**	0.059 (0.024)	**0.010**	0.067 (0.024)	**0.003**
T_1 FI-IRT → T_2 FI-IRT	0.236 (0.030)	**<0.001**	0.236 (0.046)	**<0.001**	0.213 (0.036)	**<0.001**	0.213 (0.029)	**<0.001**	0.209 (0.025)	**<0.001**

T_2 FI-IRT → T_3 FI-IRT	0.241 (0.030)	**<0.001**	0.223 (0.046)	**<0.001**	0.191 (0.036)	**<0.001**	0.219 (0.029)	**<0.001**	0.230 (0.025)	**<0.001**
Residual Correlations										
T_1: SRS ↔ SII	0.027 (0.004)	0.298	0.028 (0.004)	0.280	0.026 (0.003)	0.317	0.066 (0.006)	**0.016**	0.016 (0.004)	0.553
T_1: SRS ↔ FI-IRT	0.157 (0.001)	**<0.001**	0.192 (0.000)	**<0.001**	0.159 (0.000)	**<0.001**	0.141 (0.001)	**<0.001**	0.131 (0.001)	**<0.001**
T_1: SII ↔ FI-IRT	0.097 (0.002)	**0.001**	0.127 (0.001)	**<0.001**	0.078 (0.001)	**0.004**	0.092 (0.002)	**0.002**	0.083 (0.002)	**0.004**
T_2: SRS ↔ SII	−0.001 (0.002)	0.944	−0.001 (0.002)	0.970	0.001 (0.002)	0.947	0.028 (0.004)	0.136	−0.008 (0.003)	0.697
T_2: SRS ↔ FI-IRT	0.078 (0.001)	**<0.001**	0.115 (0.000)	**<0.001**	0.114 (0.000)	**<0.001**	0.111 (0.001)	**<0.001**	0.084 (0.001)	**<0.001**
T_2: SII ↔ FI-IRT	0.059 (0.001)	**0.002**	0.096 (0.001)	**<0.001**	0.066 (0.001)	**<0.001**	0.058 (0.001)	**0.003**	0.052 (0.001)	**0.004**
T_3: SRS ↔ SII	−0.001 (0.002)	0.944	−0.001 (0.002)	0.970	0.001 (0.002)	0.947	0.028 (0.004)	0.136	−0.008 (0.003)	0.697
T_3: SRS ↔ FI-IRT	0.087 (0.001)	**<0.001**	0.104 (0.000)	**<0.001**	0.107 (0.000)	**<0.001**	0.130 (0.001)	**<0.001**	0.092 (0.001)	**<0.001**
T_3: SII ↔ FI-IRT	0.061 (0.001)	**0.002**	0.079 (0.001)	**<0.001**	0.057 (0.001)	**<0.001**	0.060 (0.001)	**0.003**	0.054 (0.001)	**0.004**
Between-Person Relations										
RI_{SRS} ↔ RI_{SII}	0.138 (0.004)	**<0.001**	0.136 (0.004)	**0.001**	0.138 (0.004)	**<0.001**	0.123 (0.007)	**0.006**	0.136 (0.004)	**0.001**
RI_{SRS} ↔ $RI_{FI\text{-}IRT}$	0.264 (0.001)	**<0.001**	0.180 (0.001)	**0.002**	0.191 (0.000)	**<0.001**	0.225 (0.002)	**<0.001**	0.212 (0.001)	**<0.001**
RI_{SII} ↔ $RI_{FI\text{-}IRT}$	−0.054 (0.002)	0.405	−0.067 (0.001)	0.285	−0.006 (0.001)	0.923	−0.047 (0.002)	0.443	−0.064 (0.002)	0.295

Note. β = standardized coefficient; SE = standard error; P = P value, with boldface indicating statistical significance at $P < 0.05$. GRM = graded response model (primary analysis); LL = log-logistic transformation of GRM-derived frailty scores; Deficit Accumulation FI = traditional frailty index derived using an equally weighted cumulative deficit approach; MI = multiple imputation using *mice* ($m = 5$, predictive mean matching). RI = random intercept; SRS = Sarcopenia Risk Score; SII = Social Isolation Index; FI-IRT= item response theory-derived frailty index.

[a] All models were estimated using the same RI-CLPM specification, adjusting for time-varying covariates (depressive symptoms, smoking status, drinking status, and sleep quality) and time-invariant covariates (age, sex, education, and residence), with MLR as the estimator. Models 4d–6 and 8 were fitted to the same primary analytic sample generated by random-forest imputation (n = 3,159), unless otherwise specified.

[c] Participants with baseline frailty were excluded (FI-IRT ≥ 0.7038 on the 0–1 scale, corresponding to GRM $\theta \geq 1.341$ or a traditional FI of approximately 0.25); n = 2,964.

[d] MI estimates were pooled across five multiply imputed datasets using Rubin's rules. Extreme-value screening was performed after imputation; n = 3,069 in each imputed dataset.

Table S16. Bootstrap-based tests of longitudinal indirect effects in the primary and sensitivity RI-CLPMs (unstandardized estimates).

Indirect pathway	*B*	*SE*	95% BCa CI	
			Lower	Upper
Model 4d — Graded response model of the IRT-based frailty index (primary specification)				
T_1 SRS → T_2 SII → T_3 FI-IRT	0.0008*	0.0005	0.0001	0.0023
T_1 FI-IRT → T_2 SII → T_3 SRS	0.0049*	0.0025	0.0013	0.0110
Model 5 — Deficit-accumulation frailty index (FI-DA)				
T_1 SRS → T_2 SII → T_3 FI-DA	0.0004*	0.0003	0.0001	0.0012
T_1 FI-DA → T_2 SII → T_3 SRS	0.0134*	0.0074	0.0035	0.0329
Model 6 — log-logistic transformed frailty score (LL)				
T_1 SRS → T_2 SII → T_3 FI-IRT-LL	0.0003	0.0003	−0.0001	0.0009
T_1 FI-IRT-LL → T_2 SII → T_3 SRS	0.0067*	0.0038	0.0013	0.0169
Model 7 — Multiple imputation				
T_1 SRS → T_2 SII → T_3 FI-IRT	0.0007*	0.0004	0.0001	0.0016
T_1 FI-IRT → T_2 SII → T_3 SRS	0.0102*	0.0048	0.0032	0.0226
Model 8 — Exclusion of participants with baseline frailty				
T_1 SRS → T_2 SII → T_3 FI-IRT	0.0007*	0.0005	<0.0001	0.0021
T_1 FI-IRT → T_2 SII → T_3 SRS	0.0042*	0.0022	0.0009	0.0097

Note. *B* = unstandardized indirect effect (product of cross-lagged coefficients); *SE* = bootstrap standard error; BCa = bias-corrected and accelerated; 5,000 nonparametric bootstrap resamples under a stationarity-constrained RI-CLPM with ML estimation. * 95% BCa CI excludes zero. SRS, sarcopenia risk score; SII, social isolation index; FI-IRT, IRT-based frailty index; GRM, graded response model; FI-DA, deficit-accumulation frailty index; FI-IRT-LL, log-logistic-transformed GRM-derived frailty score.

SUPPLEMENTARY FIGURES

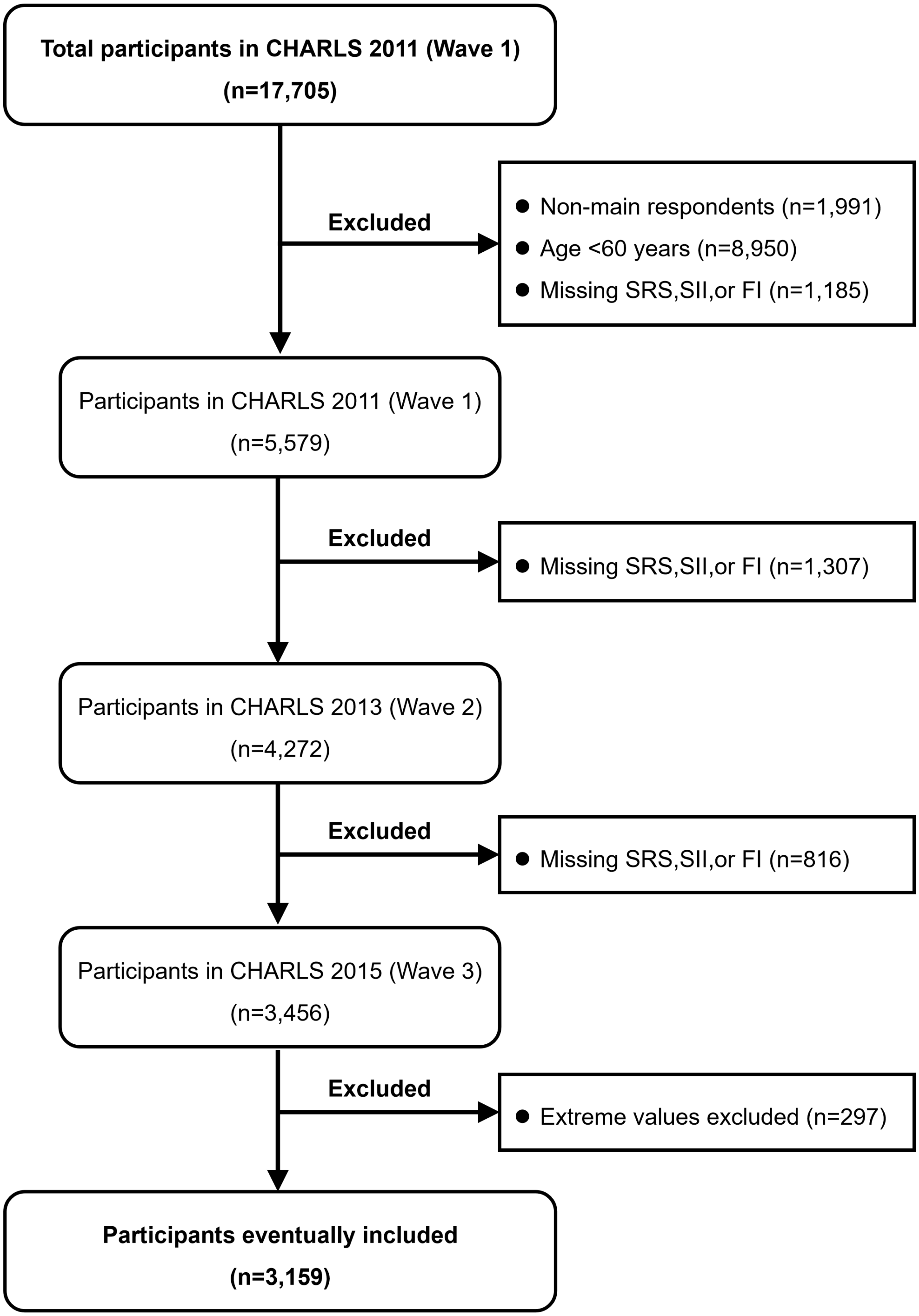

Figure S1. Participant Selection Flowchart.

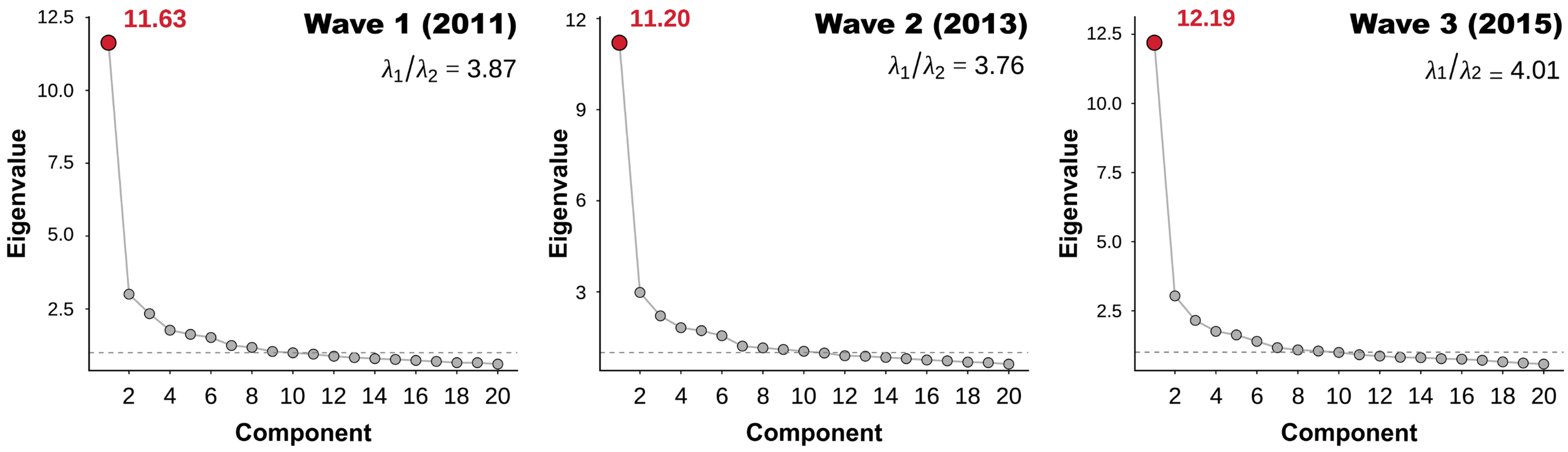

Figure S2. Scree Plots From Principal Component Analysis of the Polychoric Correlation Matrix Across Waves.

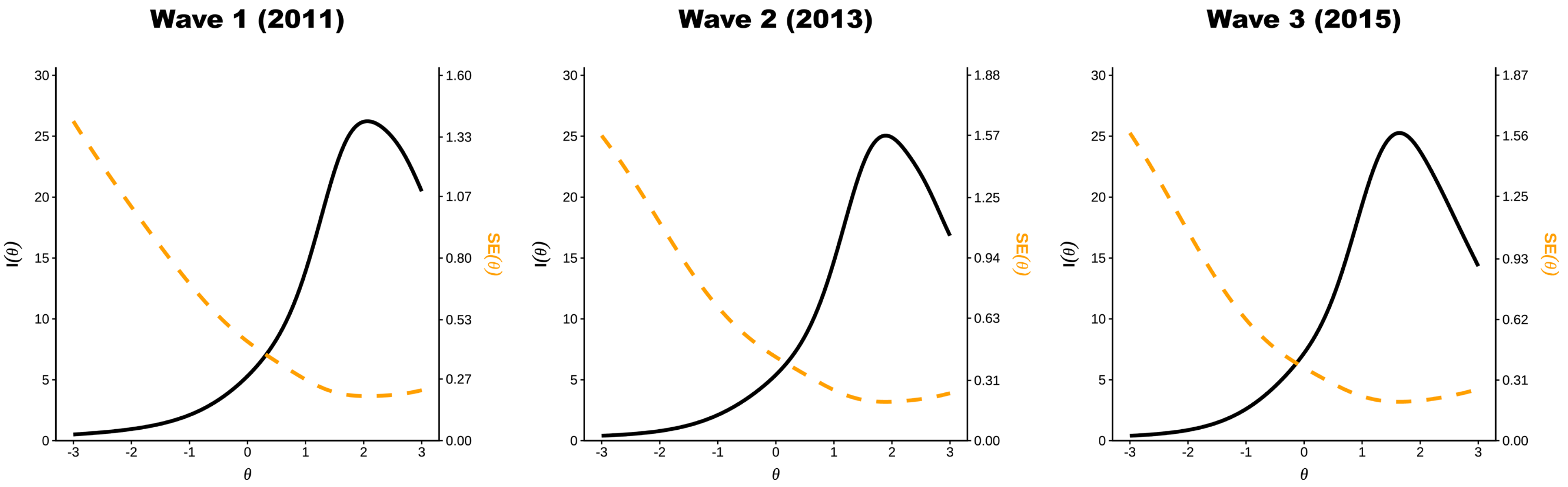

Figure S3. Test Information and Standard Error Curves for the 40-Item Frailty Index Across Waves.

Note. θ = latent frailty trait underlying the Item Response Theory-derived Frailty Index (FI-IRT).

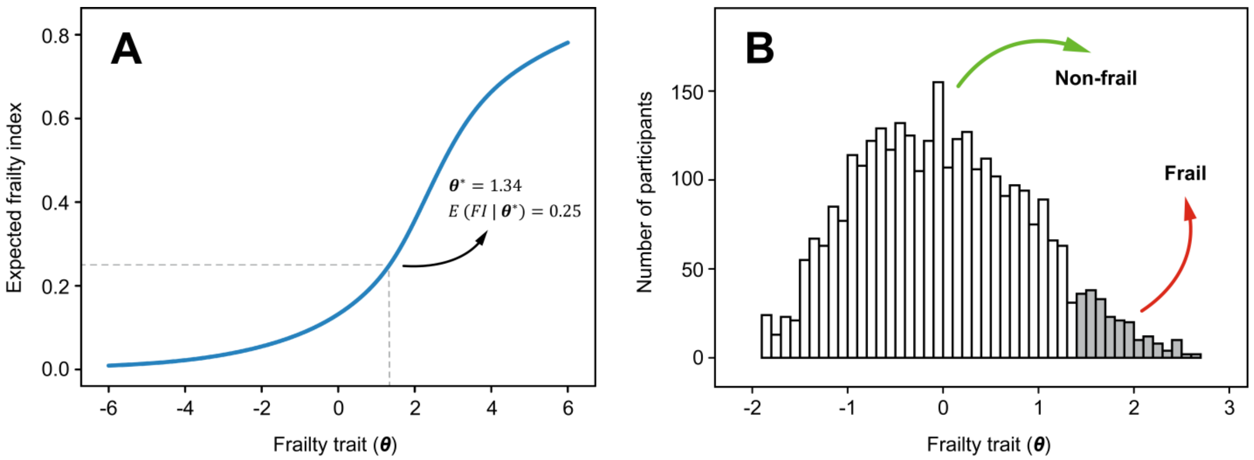

Figure S4. Test Characteristic Curve–Based Anchoring of the Frailty Threshold and Distribution of Baseline Latent Frailty Scores.

Note. (**A**) The test characteristic curve shows the expected frailty index, $E(FI \mid \theta)$, as a function of the latent frailty trait. The frailty threshold was identified over a dense θ grid from −4 to 4, and the curve is displayed over −6 to 6 for visualization. The horizontal dashed line indicates the conventional frailty threshold of 0.25 for the traditional deficit-accumulation frailty index, and the vertical dashed line indicates the corresponding latent threshold, θ^*=1.34. Their intersection defines the test characteristic curve-based threshold. (**B**) The histogram shows the distribution of baseline latent frailty scores estimated from the 40-item frailty index graded response model. Shaded bars indicate participants with $\theta \geq \theta^*$, corresponding to those classified as frail at baseline according to the test characteristic curve-derived threshold.

frontiers

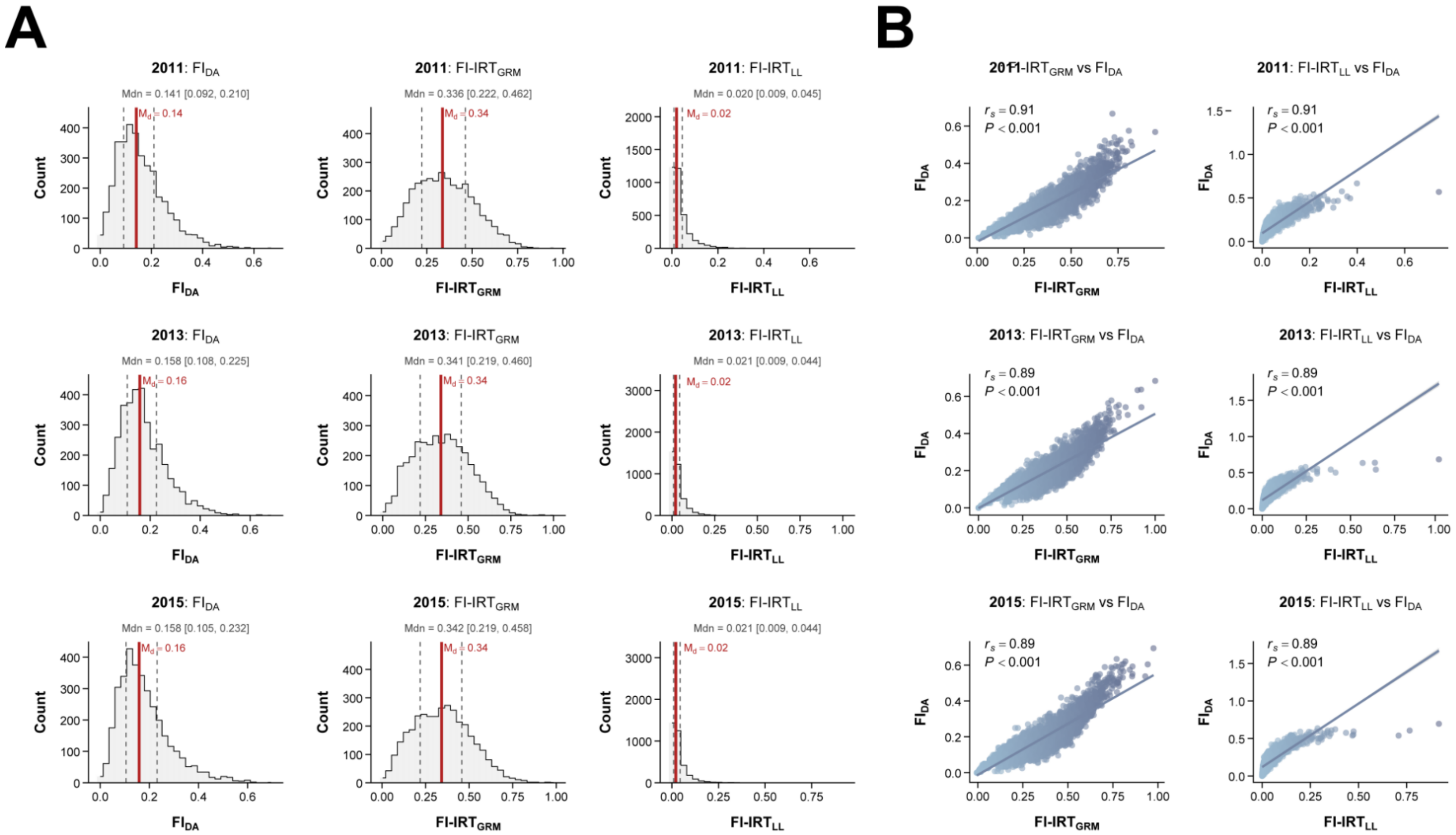

Figure S5. Distributions and Concordance of Alternative Frailty Measures Across Waves.

Note. (**A**) Histograms show the distributions of the traditional deficit-accumulation frailty index (FI-DA), the graded response model-derived item response theory frailty score (FI-IRT$_{GRM}$), and the log-logistic-transformed item response theory frailty score (FI-IRT$_{LL}$) in 2011, 2013, and 2015. In each histogram, the red solid line indicates the median, and the gray dashed lines indicate the first and third quartiles; values are shown as median [Q_1, Q_3]. (**B**) Scatter plots compare FI-IRT$_{GRM}$ and FI-IRT$_{LL}$ with FI-DA at each wave. Solid lines indicate fitted linear trends. Spearman rank correlations are shown within panels, and all correlations were statistically significant ($P < 0.001$). The figure was generated using the imputed sample before extreme outlier exclusion (n = 3,456).

Supplementary References

1. Theou O, Brothers TD, Mitnitski A, Rockwood K. Operationalization of frailty using eight commonly used scales and comparison of their ability to predict all-cause mortality. *J Am Geriatr Soc* (2013) 61:1537–1551. doi: 10.1111/jgs.12420
2. Austin PC. Balance diagnostics for comparing the distribution of baseline covariates between treatment groups in propensity-score matched samples. *Stat Med* (2009) 28:3083–3107. doi: 10.1002/sim.3697
3. Zhang Z, Kim HJ, Lonjon G, Zhu Y, Group written on behalf of AB-DCTC. Balance diagnostics after propensity score matching. *Ann Transl Med* (2019) 7:16–16. doi: 10.21037/atm.2018.12.10
4. Cohen J. *Statistical power analysis for the behavioral sciences*. 0 ed. Routledge. (2013). doi: 10.4324/9780203771587
5. Zhang Z, Gayle AA, Wang J, Zhang H, Cardinal-Fernández P. Comparing baseline characteristics between groups: An introduction to the CBCgrps package. *Ann Transl Med* (2017) 5:484–484. doi: 10.21037/atm.2017.09.39
6. Schober P, Bossers SM, Schwarte LA. Statistical significance versus clinical importance of observed effect sizes: What do P values and confidence intervals really represent? *Anesth Analg* (2018) 126:1068. doi: 10.1213/ANE.0000000000002798
7. Pulvera D. Development and validation of the sarcopenia composite index: a comprehensive approach for assessing sarcopenia in the ageing population - annals singapore. (2025) https://annals.edu.sg/development-and-validation-of-the-sarcopenia-composite-index-a-comprehensive-approach-for-assessing-sarcopenia-in-the-ageing-population/ [Accessed April 4, 2026]
8. Berkman LF, Syme SL. Social networks, host resistance, and mortality: A nine-year follow-up study of alameda county residents. *Am J Epidemiol* (1979) 109:186–204. doi: 10.1093/oxfordjournals.aje.a112674
9. Zheng H, Xiao T, Zhang M, Huang X, Chen Y, Lin Q, Xu L. Latent class analysis and determinants of social isolation among Chinese middle-aged and older adults with multimorbidity. *J Affect Disord* (2026) 399:120793. doi: 10.1016/j.jad.2025.120793
10. Samejima F. Estimation of latent ability using a response pattern of graded scores. *Psychometrika* (1969) 34:1–97. doi: 10.1007/BF03372160
11. Wignall L, McCormack ,Mark, Carpino ,Tom, Owens ,Rebecca, and Barton T. The kink orientation scale: Developing and validating a measure of kink desire, practice, and identity. *J Sex Res* (2025) 62:307–316. doi: 10.1080/00224499.2024.2387769
12. Gao X, Liu Z. Analyzing the psychometric properties of the PHQ-9 using item response theory in a chinese adolescent population. *Ann Gen Psychiatry* (2024) 23:7. doi: 10.1186/s12991-024-00492-3
13. Smits N, Cuijpers P, Van Straten A. Applying computerized adaptive testing to the CES-D scale: a simulation study. *Psychiatry Res* (2011) 188:147–155. doi: 10.1016/j.psychres.2010.12.001
14. Maydeu-Olivares A. Goodness-of-fit assessment of item response theory models. *Meas: Interdiscip Res Perspect* (2013) https://www.tandfonline.com/doi/abs/10.1080/15366367.2013.831680 [Accessed July 4, 2025]
15. Pellicciari L, Chiarotto A, Giusti E, Crins MHP, Roorda LD, Terwee CB. Psychometric properties of the patient-reported outcomes measurement information system scale v1.2: global health (PROMIS-GH) in a Dutch general

population. *Health Qual Life Outcomes* (2021) 19:226. doi: 10.1186/s12955-021-01855-0

16. Millsap RE. Testing measurement invariance using item response theory in longitudinal data: An introduction. *Child Dev Perspect* (2010) 4:5–9. doi: 10.1111/j.1750-8606.2009.00109.x

17. Maydeu-Olivares A, Joe H. Limited information goodness-of-fit testing in multidimensional contingency tables. *Psychometrika* (2006) 71:713–732. doi: 10.1007/s11336-005-1295-9

18. A new statistic for evaluating item response theory models for ordinal data. *CRESST* https://cresst.org/publication/a-new-statistic-for-evaluating-item-response-theory-models-for-ordinal-data/ [Accessed June 3, 2026]

19. Chen FF. Sensitivity of goodness of fit indexes to lack of measurement invariance. *Struct Equ Model: Multidiscip J* (2007) 14:464–504. doi: 10.1080/10705510701301834

20. Buchholz J, Hartig J. Comparing attitudes across groups: An IRT-based item-fit statistic for the analysis of measurement invariance. *Appl Psychol Meas* (2019) 43:241–250. doi: 10.1177/0146621617748323

21. Tay L, Meade AW, Cao M. An overview and practical guide to IRT measurement equivalence analysis. *Organ Res Methods* (2015) 18:3–46. doi: 10.1177/1094428114553062

22. Engels O, Lüdtke O, Robitzsch A. Estimating trends with differential item functioning: A comparison of five IRT-based approaches. *Educ Psychol Meas* (2026)131644251408818. doi: 10.1177/00131644251408818

23. Köhler C, Robitzsch A, Hartig J. A bias-corrected RMSD item fit statistic: An evaluation and comparison to alternatives. *J Educ Behav Stat* (2020) 45:251–273. doi: 10.3102/1076998619890566

24. Robitzsch A, Lüdtke O. Mean comparisons of many groups in the presence of DIF: An evaluation of linking and concurrent scaling approaches. *J Educ Behav Stat* (2022) 47:36–68. doi: 10.3102/10769986211017479

25. Terluin B, Koopman JE, Hoogendam L, Griffiths P, Terwee CB, Bjorner JB. Estimating meaningful thresholds for multi-item questionnaires using item response theory. *Qual Life Res* (2023) 32:1819–1830. doi: 10.1007/s11136-023-03355-8

26. Pua Y-H, Terluin B, Tay L, Clark RA, Thumboo J, Tay E-L, Mah S-M, Ng Y-S. Using item response theory to estimate interpretation threshold values for the frailty index in community dwelling older adults. *Arch Gerontol Geriatr* (2024) 117:105280. doi: 10.1016/j.archger.2023.105280

27. Rockwood K, Andrew M, Mitnitski A. A comparison of two approaches to measuring frailty in elderly people. *J Gerontol: A* (2007) 62:738–743. doi: 10.1093/gerona/62.7.738

28. Song X, Mitnitski A, Rockwood K. Prevalence and 10-year outcomes of frailty in older adults in relation to deficit accumulation. *J Am Geriatr Soc* (2010) 58:681–687. doi: 10.1111/j.1532-5415.2010.02764.x

29. Buuren S van, Groothuis-Oudshoorn K. mice: Multivariate imputation by chained equations in R. *J Stat Softw* (2011) 45:1–67. doi: 10.18637/jss.v045.i03

30. R: the R project for statistical computing. https://www.r-project.org/ [Accessed April 22, 2026]

31. Chalmers RP. mirt: A multidimensional item response theory package for the R environment. *J Stat Softw* (2012) 48:1–29. doi: 10.18637/jss.v048.i06

32. Revelle W. psych: procedures for psychological, psychometric, and personality research. (2026) https://cran.r-project.org/web/packages/psych/index.html [Accessed April 22, 2026]

33. Mayer M. missRanger: Fast imputation of missing values. (2024) https://cran.r-project.org/web/packages/missRanger/index.html [Accessed November 14, 2025]

34. Rosseel Y, Jorgensen TD, Wilde LD, Oberski D, Byrnes J, Vanbrabant L, Savalei V, Merkle E, Hallquist M, Rhemtulla M, et al. lavaan: latent variable analysis. (2025) https://cran.r-project.org/web/packages/lavaan/index.html [Accessed April 22, 2026]

35. Buuren S van, Groothuis-Oudshoorn K, Vink G, Schouten R, Robitzsch A, Rockenschaub P, Doove L, Jolani S, Moreno-Betancur M, White I, et al. mice: multivariate imputation by chained equations. (2025) https://cran.r-project.org/web/packages/mice/index.html [Accessed April 22, 2026]
